# Defect-Driven Efficient Selective CO_2_ Hydrogenation with Mo-Based Clusters

**DOI:** 10.1021/jacsau.3c00206

**Published:** 2023-09-15

**Authors:** Jiajun Zhang, Kai Feng, Zhengwen Li, Bin Yang, Binhang Yan, Kai Hong Luo

**Affiliations:** † National Engineering Research Center of Green Recycling for Strategic Metal Resources, Institute of Process Engineering, Chinese Academy of Sciences, Beijing 100190, China; ‡ Center for Combustion Energy, Key Laboratory for Thermal Science and Power Engineering of Ministry of Education, International Joint Laboratory on Low Carbon Clean Energy Innovation, Tsinghua University, Beijing 100084, China; § Department of Chemical Engineering, Tsinghua University, Beijing 100084, China; ∥ Department of Mechanical Engineering, University College London, Torrington Place, London WC1E 7JE, U.K.

**Keywords:** Mo_2_C nanoparticle, synthesized fuels, RWGS reaction, atomic magnetism, selective hydrogenation

## Abstract

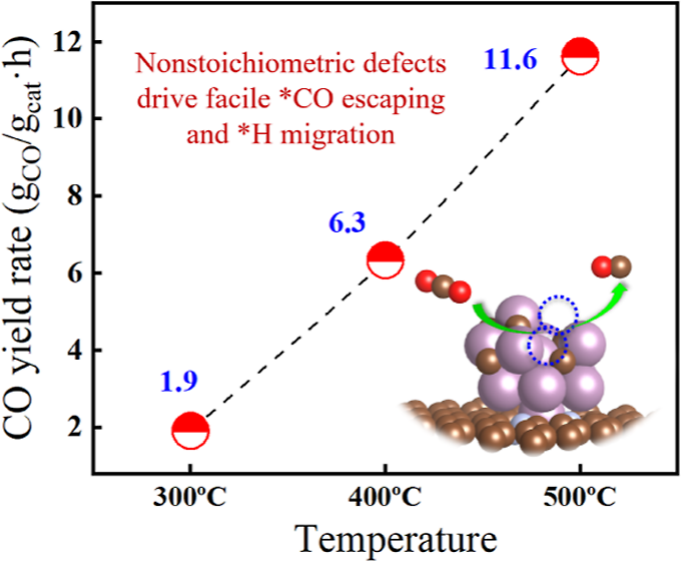

Synthetic fuels produced from CO_2_ show promise in combating climate change. The reverse water gas shift (RWGS) reaction is the key to opening the CO_2_ molecule, and CO serves as a versatile intermediate for creating various hydrocarbons. Mo-based catalysts are of great interest for RWGS reactions featured for their stability and strong metal–oxygen interactions. Our study identified Mo defects as the intrinsic origin of the high activity of cluster Mo_2_C for CO_2_-selective hydrogenation. Specifically, we found that defected Mo_2_C clusters supported on nitrogen-doped graphene exhibited exceptional catalytic performance, attaining a reaction rate of 6.3 g_CO_/g_cat_/h at 400 °C with over 99% CO selectivity and good stability. Such a catalyst outperformed other Mo-based catalysts and noble metal-based catalysts in terms of facile dissociation of CO_2_, highly selective hydrogenation, and nonbarrier liberation of CO. Our study revealed that as a potential descriptor, the atomic magnetism linearly correlates to the liberation capacity of CO, and Mo defects facilitated product desorption by reducing the magnetization of the adsorption site. On the other hand, the defects were effective in neutralizing the negative charges of surface hydrogen, which is crucial for selective hydrogenation. Finally, we have successfully demonstrated that the combination of a carbon support and the carbonization process synergistically serves as a feasible strategy for creating rich Mo defects, and biochar can be a low-cost alternative option for large-scale applications.

## 1 Introduction

CO_2_ hydrogenation to produce synthetic fuels is an important strategy to realize the goal of “net zero” emission based on existing energy infrastructures.^1^ CO is an important intermediate from CO_2_ hydrogenation and can be used directly as fuel or as feedstock in tandem reactions for a diverse range of fuel products.^2^ A variety of noble metal-based catalysts have been developed for reverse water gas shift (RWGS) reactions in recent years,^3–8^ but their high cost poses insurmountable challenges for commercial implementation. Non-noble metals for the RWGS reaction normally have poor activity and selectivity, especially at low temperatures, but there are exceptions, being highly dependent on the fine regulation of the local electronic structures for the active sites.^9–13^

For example, Co-based catalysts are always deemed to fail to catalyze the RWGS reaction,^14^ but the nitrogen coordinated single atom Co performed almost 100% selectivity to CO with over 50% conversion during CO_2_ hydrogenation, with a gas hourly space velocity (GHSV) of 6000 mL/(g h) and a carbon-to-hydrogen ratio of 4:1, providing a new strategy for designing base metal-based RWGS catalysts.^15^ CeO_2_ with surface regulation also exhibited a high activity in the RWGS reaction. Dedicated oxygen defects on the catalysts realized a balance between CO_2_ dissociation and surface hydrogenation, delivering the best activity.^16^ Native point defects in vanadium carbide strengthened the electronic interactions between the reactant and the catalyst, facilitating the RWGS reaction.^17^ Tailored multicomponent catalysts, including Cu and Fe alloys, were also commonly seen to promote the RWGS performance,^18^ where Cu promotes CO_2_ activation, and normally leads to the formate mechanism for the hydrogenation, while Fe improves the thermal stability and was found to favor redox mechanism for hydrogenation. Alkali metals were frequently doped to improve CO_2_ adsorption and suppress excess adsorption of H_2_, leading to methanation.^19,20^

Mo-based catalysts have shown outstanding performance in the RWGS reaction among non-noble catalysts. Molybdenum carbides (Mo_2_C), nitrides (Mo_2_N), phosphides (MoP), and metallic molybdenum (Mo) are of particular interest for the selective hydrogenation of CO_2_. They are favored due to their ability to form strong metal–oxygen bonds and stability, which are crucial for the RWGS reaction.^21^ Nanoparticles have been shown to have better catalytic performance compared to extended surfaces in terms of adsorption and dissociation of CO_2_, as well as hydrogen diffusion.^22,23^

MoP supported on SiO_2_ has shown fully CO-selective capability at 450 °C and was shown to remain stable for over 22 h.^24^ Hexagonal 2D-Mo_2_C catalysts exhibited higher activity than β-Mo_2_C for CO formation by a rate of roughly 1180 mg/h/g_cat_ at 230 °C, with a H_2_ to CO_2_ ratio of 3:1. This was benefited from the unsaturated surface attained during the preparation from Mo_2_CT_*x*_ with abundant surface termination groups of O, OH, and F.^25^ Mo_2_N has also been found to be active and outperform W_2_N and NbN for CO_2_ hydrogenation with high CO selectivity. The interstitial vacancies in the crystal could be reversibly filled with the oxygen, facilitating CO_2_ dissociation and hydrogenation through the redox mechanism.^26^ With the addition of Pt, MoO_3_ was found to form MoO_*x*_-rich O vacancies over Mo_2_N, and the metal-vacancy synergistic sites were much more active for the RWGS reaction. The catalyst realized nearly 20% conversion in a high GHSV of 3,000,000 mL/g/h with a carbon-to-hydrogen ratio of 1:3.^27^ The insertion of N into the interstitial site of CoMo alloy was also observed to promote the catalytic performance for the RWGS reaction. H_2_ experiences dissociative adsorption on N, and Mo becomes more electron deficient, such that it is beneficial for CO desorption.^28^ A single-atom catalyst of Mo coordinated by N demonstrated very high selectivity to CO due to the lack of an atomic ensemble. It gives rise to exothermic desorption of product molecules even at low temperature of 300 °C, and the conversion of CO_2_ reached 24% at 500 °C with a C/H ratio of 1:3.^29,30^

Mo-based catalysts, including their alloys, have attracted significant interest in recent years due to their high performance in selective hydrogenation. However, the origin of their activity, especially for cluster catalysts, has not been fully understood, despite numerous efforts to establish more efficient low-temperature catalytic systems for the RWGS reaction. This study proposed that Mo defects are the decisive factor for the superior activity of Mo_2_C cluster in the selective hydrogenation to CO_2_. Based on this, a facile strategy is demonstrated to improve the intrinsic catalytic activity. We also report on the mechanisms underlying the high activity of the defected Mo clusters, including defect-induced neutralization of surface H charges to promote selective hydrogenation, and the affinity between product desorption capacity and defect-induced atomic magnetization. These mechanisms are supported by multiscale modeling results and experimental evidence.

## 2 Results and Discussion

### 2.1 Performance of Mo-Based Catalysts under RWGS Conditions

**Figure 1:**
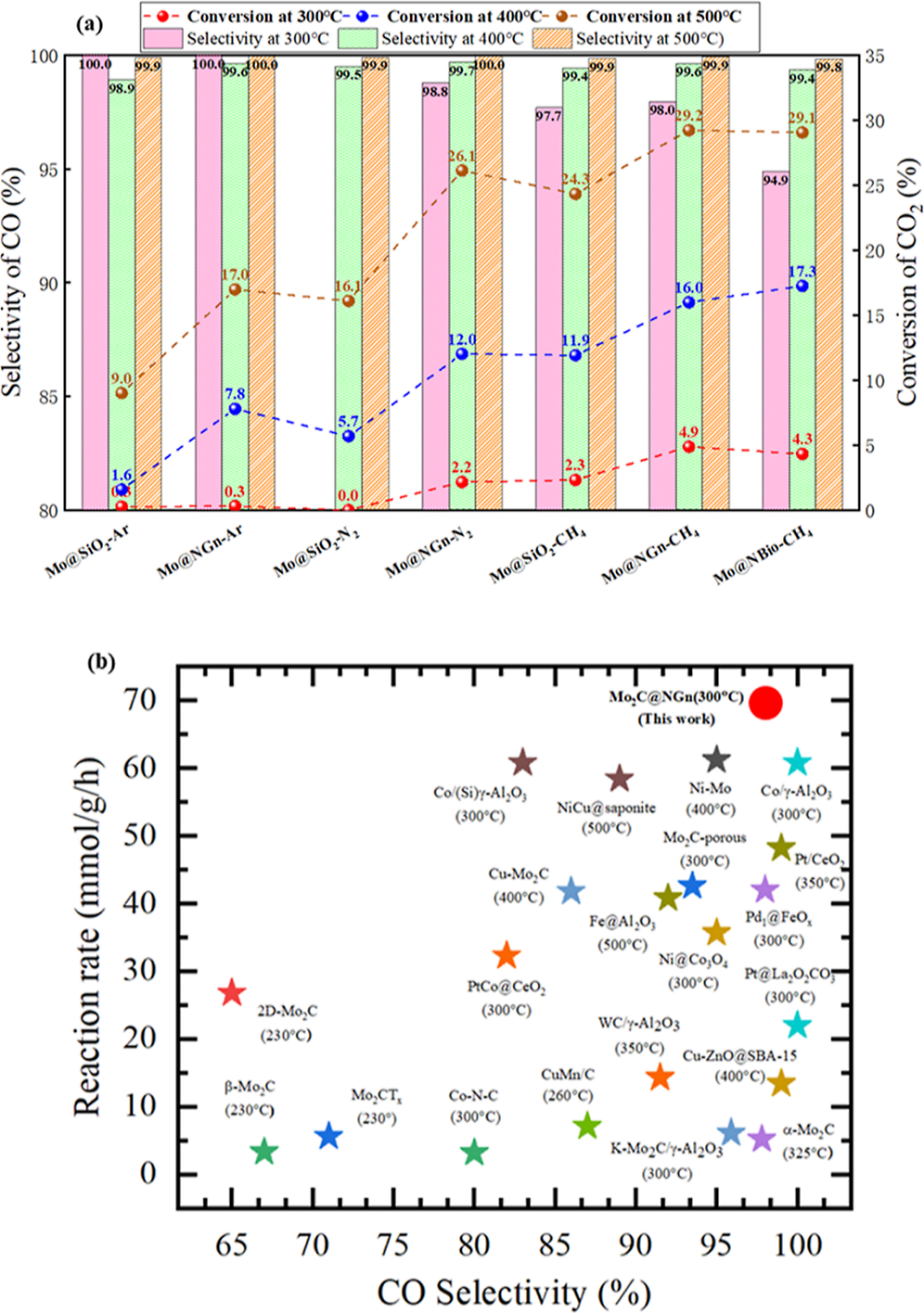
(a) Conversion of CO_2_ and selectivity of CO for the catalysts of the Mo support on carbon and SiO_2_ pretreated under reduction, nitrogenation, and carbonization, respectively, at 300 °C/400 °C/500 °C (GHSV = 140,000 mL/g/h, mol_CO_2__/mol_H_2__ = 1:2). (b) Summary of the performance under RWGS conditions for the reported catalysts.

The catalytic performance of a variety of Mo-based catalysts was evaluated at 300, 400, and 500 °C in a sequential test. Specifically, we compared Mo-based cluster catalysts prepared in the atmosphere of argon and hydrogen (reduction, Mo), nitrogen and hydrogen (nitrogenation, Mo_2_N), and methane and hydrogen (carbonization, Mo_2_C), respectively. Supports of nitrogen-doped graphene (NGn), nitrogen-doped biochar (NBiochar), and SiO_2_ were tested to reveal their effects on the catalytic performance, as shown in Figure 1a.

We observed that the conversion of CO_2_ generally increased with temperature, and the catalysts pretreated by carbonization always gave rise to better catalytic activity. The conversion of CO_2_ reached 24.3% over Mo@SiO_2_-CH_4_ at 500 °C, 29.2% over Mo@NGn-CH_4_ at the same temperature, and 16.0% at 400 °C and 4.9% at 300 °C. The peak conversion for the nitrogenized Mo@NGn was 26.1% at 500 °C and only 17.0% for the reduced catalyst. The selectivity of CO in the RWGS reaction was almost 100% at 500 °C and not much affected by the catalyst preparation atmosphere. Only slight changes caused by temperature were observed for the nitrogenized catalysts and carbonized catalysts. In contrast, we observed the non-negligible effects of the support on catalytic activity; carbon support gave rise to better catalytic activity compared to SiO_2_, wherein the nitrogen doping may play a key role in stabilizing the metal-based nanoparticles.^31–33^ By comparing the nitrogenized Mo@NGn and Mo@SiO_2_, a 10.0% higher conversion was observed at 500 °C and a 6.3% rise at 400 °C. For the catalysts prepared in methane, the conversion over the carbon support was about two-fold of that over Mo@SiO_2_ at 300 °C. We concluded from the experimental data that the Mo-based catalysts prepared under the carbonization conditions result in the best catalytic performance for the RWGS reaction, and carbon supports would further promote the catalytic activity.

The normalized catalytic performance of reported metal-based catalysts under the low-temperature RWGS conditions was compared with Mo@NGn-CH_4_ and is shown in Figure 1b and Table S1. We observed that Mo@NGn-CH_4_ is especially outstanding in terms of catalytic activity and CO selectivity among recently reported single-metal-based catalysts and even noble metal-based catalysts,^25,34^ and its low-temperature performance is superior compared to other Mo family catalysts including α/β-Mo_2_C, 2D-Mo_2_C, and porous Mo_2_C, attaining a CO_2_ reaction rate of 69 mmol/g/h and a CO selectivity of 98% (yield rate of 1904 mg_CO_/g/h) at 300 °C, and a reaction rate of 227 mmol/g/h and selectivity of 99.6% (yield rate of 6331 mg_CO_/g/h) at 400 °C, and 415 mmol/g/h for reaction rate and 99.9% for selectivity (yield rate of 11,620 mg_CO_/g/h) at 500 °C, indicating high atom usage efficiency of Mo_2_C@NGn. We also prepared biochar as an alternative carbon support, which has been found to give rise to CO_2_ conversion as NGn. Despite slightly lower selectivity of CO at 300 °C, biochar finds its preponderance of benefit in terms of economy.

**Figure 2:**
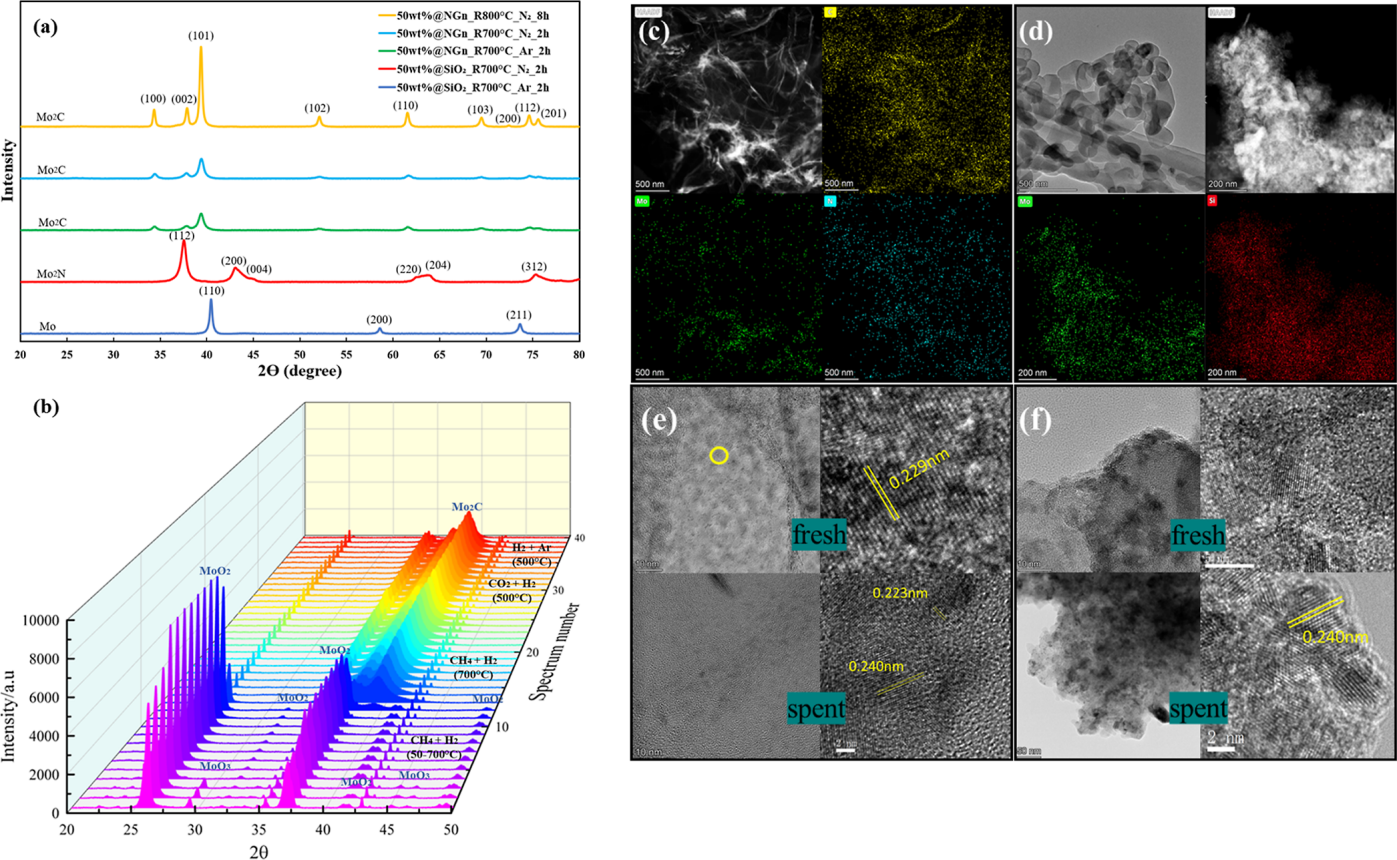
(a) XRD spectra of the Mo-based catalysts. (b) In situ XRD spectra of Mo@NGn during carbonization, RWGS reaction, and reductive treatment. HAADF images and EDS elemental mapping images of (c) fresh Mo@NGn-CH_4_ and (d) Mo_2_C@SiO_2_-CH_4_. HD-TEM images for fresh and spent catalysts (e) Mo_2_C@NGn-CH_4_ and (f) Mo_2_C@SiO_2_-CH_4_.

To clearly identify the active species on the catalysts, we implemented X-ray diffraction (XRD) tests on the above catalysts. The results in Figure 2a reveal that no matter what treatment atmosphere was applied during catalyst preparation over the NGn support, Mo_2_C is the actual active site for all the catalysts supported on carbon, as evidenced by the characteristic peaks at 34.3, 37.7, 39.3, 51.9, 61.5, 69.1, 72.4, 74.5, and 75.5° for (1 0 0), (0 0 2), (1 0 1), (1 0 2), (1 1 0), (1 0 3), (2 0 0), (1 1 2), and (2 0 1) facets for C_1.02_Mo_1.98_, respectively (PDF#65-8364).^35–37^ Besides, the results also reveal that longer treatment and higher temperature contributed to a higher peak intensity of Mo_2_C, implying that the interaction between the carbon support and Mo is strengthened with a larger size of clusters. To verify the carbon support–metal interaction, we implemented additional XRD tests to the SiO_2_-supported catalysts of Mo@SiO_2_-N_2_ and Mo@SiO_2_-Ar. Not surprisingly, we merely observed the characteristic peaks at 37.7 and 43.0° for Mo_2_N (1 1 2) and (2 0 0) for Mo@SiO_2_-N_2_ (PDF#75-1150), and the peaks at 40.5, 58.7, and 73.7° are of metallic Mo (1 1 0), (2 0 0), and (2 1 1), respectively, for Mo@SiO_2_-Ar (PDF#89-5023),^38,39^ as shown in Figure 2a. Considering the surface reaction results, we confirm that Mo_2_C has superior catalytic behavior over Mo_2_N or Mo in the RWGS reaction and that the carbon support is vital for Mo_2_C formation in the carbonization process. We then carried out an in situ XRD test to Mo@NGn-CH_4_ (known as Mo_2_C@NGn) to reveal its full-life evolution, as shown in Figure 2b, and the corresponding species for the main peaks are labeled. During the carbonization period, main characteristic peaks of the catalyst precursor were found, namely, the peak at 26.0° for MoO_2_ (1 1 0) and 36.9° for MoO_2_ (0 2 0), together with some other small peaks at 31.7, 41.3, and 49.4° (PDF#73-1249).^40^ Besides, the peaks of 29.4, 45.4, 46.6, and 48.9° are identified as MoO_3_ (PDF#21-0569).^41^ After Mo_2_C formation, it was identified with the same peaks as those identified in the ex situ XRD test. Besides, some constant tiny peaks at 25.6, 35.1, 42.7, and 43.2° were also noticed, and they are identified to be the component material (Al_2_O_3_) of the crucible exposed to the X-ray during the text (PDF#75-1864 & PDF#34-0493).^42,43^ During the two-stage carbonization process in the CH_4_ and H_2_ atmosphere, we observed an intensity increase for the precursor peaks before being fully eliminated, indicating growth of the particle size of the metal oxide particles which may experience a redispersion during the carbonization process.^44^ A constant signal for Mo_2_C appeared at nearly 700 °C at the end of the heating up stage. The results also revealed that Mo_2_C active site remained stable throughout the RWGS reaction conditions at 500 °C, without any oxidation or phase changes.^45^ When applying only H_2_ and Ar instead of the reactive atmosphere, we found that Mo_2_C@NGn would still remain stable in such a reductive atmosphere, further confirming the stability of the active site. From the above tests, we can also confirm that Mo_2_C was the sole active site in our experiments with CO_2_ hydrogenation. Transmission electron microscopy (TEM) was adopted to reveal the appearance of the as-obtained Mo_2_C-based catalysts and spent catalysts. The high-angle annular dark-field (HAADF) image in Figure 2c demonstrated film-like structure of the graphene-supported catalyst. Mapping images by energy-dispersive X-ray spectroscopy (EDS) for Mo_2_C@NGn show the homogeneous distribution of Mo on NGn. Figure 2d shows the global appearance and EDS mapping images of Mo_2_C@SiO_2_. Similar distributions of Mo species were observed on the SiO_2_ support. Besides, high-resolution (HD) TEM images in Figure 2e,f show Mo_2_C species on the respective NGn and SiO_2_ support before and after the RWGS reaction, indicating the chemical stability of the active species throughout the reaction. We also observed that the Mo_2_C particles over NGn show diameters ranging from 1 to 5 nm, smaller than those over SiO_2_ (Figure S2).

**Figure 3:**
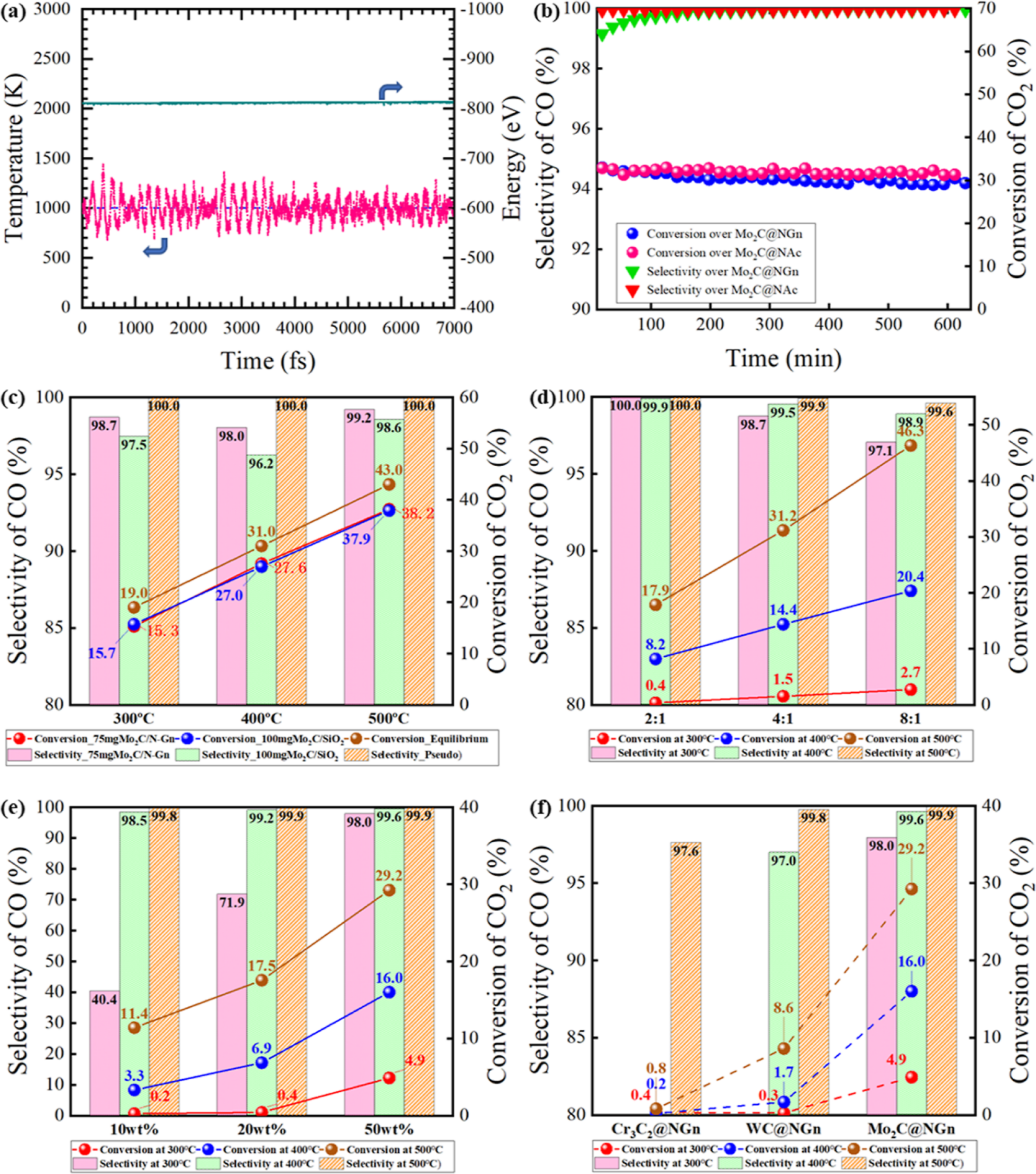
(a) Stability of the active site for Mo_2_C@NGn evaluated by AIMD at 800 °C. (b) Stability of the Mo_2_C@NGn catalyst under RWGS reaction conditions at 500 °C. The performance of Mo_2_C@NGn with various (c) temperatures (GHSV = 16,000 and 12,000 mL/g/h for Mo_2_C@NGn and Mo_2_C@SiO_2_ respectively, mol_CO_2__/mol_H_2__ = 1:2). (d) Hydrogen to carbon ratios in hydrogenation (GHSV = 318,000 mL/g/h). (e), Loading ratios. (f) VIB metal carbides under RWGS conditions (GHSV = 140,000 mL/g/h, mol_CO_2__/mol_H_2__ = 1:2 for e,f).

As the most active catalyst, the stability of Mo_2_C@NGn was directly evaluated by respective DFT modeling and catalytic performance under RWGS conditions. The cluster model (as shown in Figure S3a) was established and simulated at 800 °C by ab initio molecule dynamics (AIMD) for 7 ps, and the total enthalpy and temperature were monitored and are demonstrated in Figure 3a. The results indicate that the cluster active site remains dynamically stable under the high temperature throughout the modeling period, where doped nitrogen may play a key role in stabilizing the cluster particle.^46,47^ The fresh catalyst was tested in an experiment under the RWGS reaction conditions at 500 °C for over 10 h. Results reveal that CO selectivity and conversion of CO_2_ could both keep steady at nearly 100 and 30%, respectively. Besides, we also found from the test that the cluster supported on biochar could retain the same stability as the graphene-supported catalyst (Figure 3b). The stability of the catalyst is also confirmed by the strong interaction between the Mo_2_C cluster and NGn observed in the DFT modeling (Figure S3c), as well as the highly dispersed small particles observed in the TEM images.

We then implemented further reaction studies to clearly demonstrate the effect of the active site–support interaction on the low temperature catalytic performance. The results in Figure 3c indicate the conversion of CO_2_ over Mo_2_C@NGn reached 15.3% at 300 °C (38.2% at 500 °C and 27.6% at 400 °C) with a GHSV of 16,000 mL/h/g, approaching the conversion of chemical equilibrium, and the selectivity of CO is all above 98%. The same conversion needed 30 wt % more Mo_2_C@SiO_2_ with lower selectivity, indicating the superior synergistic effect between the carbon support and Mo. More experiments were carried out to reveal the effects of the H_2_ to CO_2_ ratio on the hydrogenation over Mo_2_C@NGn (Figure 3d), where the GHSV was kept constant for these three tests. The results show that the catalyst maintains a high CO selectivity of 97% at 300 °C even under a H-to-C ratio of 8:1, implying the outstanding capacity of the catalyst in selective hydrogenation in RWGS reactions. We have also probed the effects of the metal loading ratio on the NGn support. Figure 3 confirms that the catalytic performance is in positive correlation with the loading ratio of Mo before 50 wt %. Other group-VIB-metal-based catalysts of Cr_3_C_2_ and WC were tested under identical RWGS reaction conditions (Figure 3f). The corresponding nitrogenized catalysts were also investigated in a variety of working conditions (Figure S4). The results confirm the exceptional catalytic performance of Mo_2_C@NGn in all of the tested RWGS conditions.

### 2.2 Mechanism for Efficient RWGS Reaction over Mo_2_C@NGn

**Figure 4:**
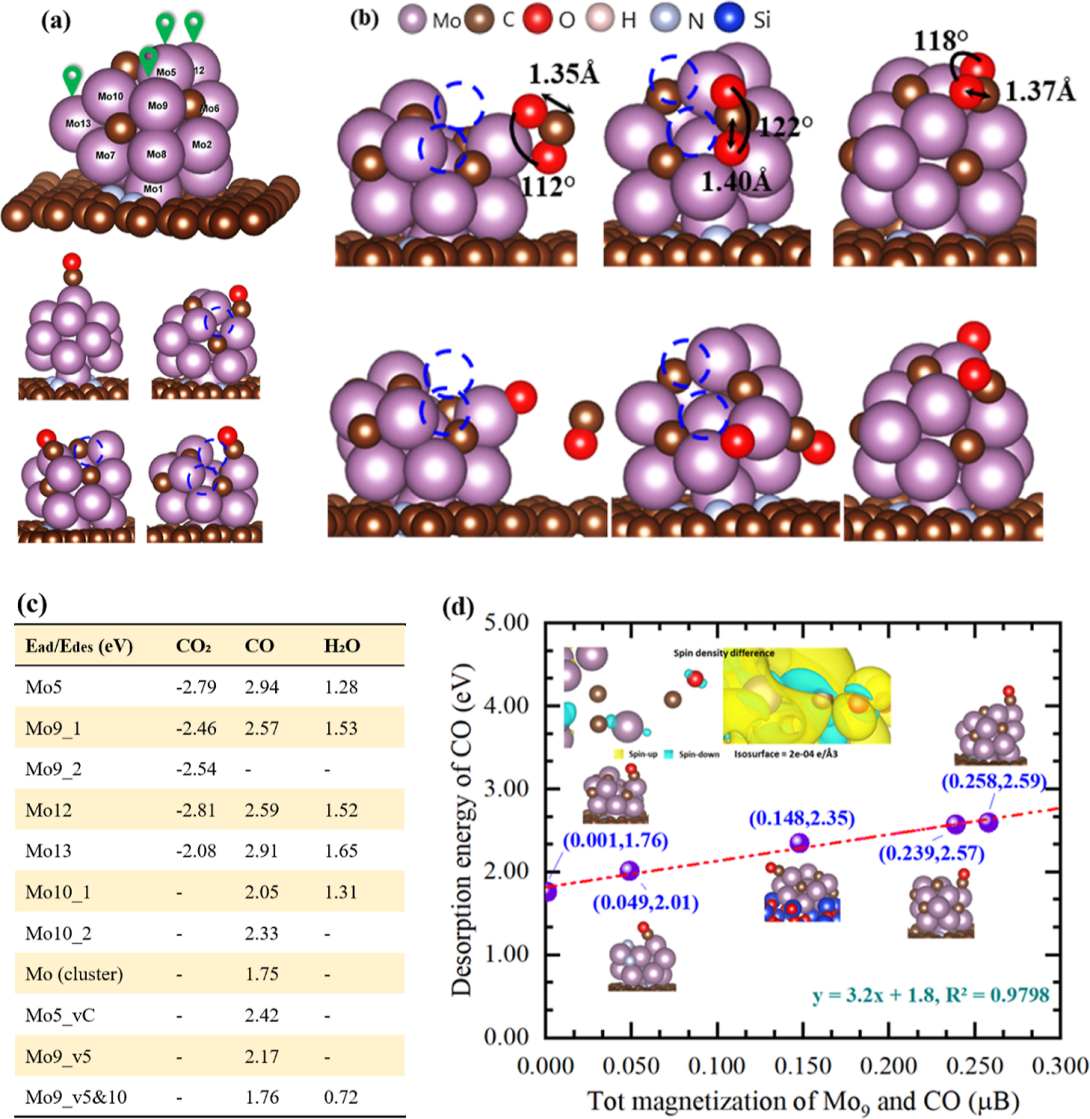
a) Models of the intact cluster and adsorption configurations of CO on Mo_2_C@NGn with various types of Mo defects (in blue dashed line circles). (b) Adsorption configurations of CO_2_ on the defected Mo_2_C@NGn and intact catalyst. (c) Summary of the adsorption energy of CO_2_, CO, and H_2_O. (d) Magnetization (net spin) of the adsorption site atom as a function of and desorption energy of CO.

The underlying mechanism of the selective hydrogenation over Mo_2_C@NGn was investigated with DFT modeling. The intact cluster model is demonstrated in Figure 4a. Potential adsorption sites to the electrophilic attack on intact clusters (labeled in green) were identified by Kukui analysis (Figure S5). The adsorption energies of CO_2_ and the desorption energies of CO and H_2_O on the potential active sites were calculated. The adsorption energies for the most stable configuration on each site are shown in Figure 4c. The results reveal CO desorption energy on intact Mo_2_C cluster ranging from 2.05 to 2.94 eV, and the corresponding CO_2_ adsorption energy ranging from −2.08 to −2.81 eV, indicating these sites have strong binding with the product molecule and hamper the RWGS reaction, which was also observed in recent study.^48,49^ We then introduced defects into the adsorption cluster, namely, one C defect and one Mo defect, respectively, on Mo_2_C (Figure 4a), the binding of the CO molecule and the cluster is slightly reduced, with a desorption energy of 2.42 and 2.17 eV, respectively. When two Mo defects were introduced to the cluster (Mo5 and Mo10, Figure 4a), we surprisingly observed a sharp decrease of the desorption energy from 2.57 to 1.76 eV for CO on the identical site (Mo9), and the site becomes the most vulnerable site to the electrophilic attach based on the prediction by Fukui analysis (Figure S6). Similarly, the desorption energy of H_2_O on such a site has also been decreased to 0.72 eV. In addition, our DFT results also predicted the CO desorption on an intact metallic Mo@NGn had a similarly small energy of 1.75 eV. These results imply that Mo_2_C with dual Mo defects as well as Mo cluster would potentially benefit products escaping during the RWGS reaction.

To reveal the underlying mechanism for the positive effects of the dual Mo defects on CO desorption, the bond strengths between adsorbate and various active sites were first considered, and Mo_2_N_vMo@NGn and Mo_2_C_vMo@SiO_2_ with dual Mo defects were included in the analysis. Surprisingly, the results in Figure S7 reveal that desorption energy has little correlations with the Mo–CO bond strength. Nevertheless, we observed significant changes of the local electronic spin (magnetization) before and after the CO adsorption onto Mo_2_C_vMo@NGn, although the net spin for the whole system is negligible throughout the desorption, and the magnetization for the adsorption site (Mo9) is also nearly zero during the interaction. In a stark contrast, Mo_2_C_vMo@SiO_2_ was nonmagnetic but the magnetization reached 1.340 μB after the CO adsorption, in which the magnetization for Mo9 saw an obvious rise to 0.123 μB as shown in Figure S8. Density of states analysis to atom Mo9 in the defected cluster and C in the CO molecule firmly verifies the origin of magnetic characteristic of Mo_2_C_vMo supported on NGn and SiO_2_, respectively, after CO adsorption (Figure S8). We then performed more magnetic analysis toward other structures, and the results in Figure 4d point out a positive linear correlation between the desorption energy of CO with the total magnetization density of the binding components of Mo9 and CO, where the major spin is indeed contributed by Mo.

Significantly, our findings reveal that the net spin for M9 is virtually negligible in all of the fresh catalysts, as is the case with the CO molecule. However, varying extents of spin polarization are introduced by the adsorption process. We posit that this is related to the charge transfer from Mo to CO, albeit in small quantities,^50^ as suggested by the partial density of states for C and Mo depicted in Figure S8, indicating that the adsorption energy is predominantly contributed by ionic-like binding. We discovered that the magnetization density at the adsorption site effectively characterizes these interactions and aligns well with the CO desorption energy.^51,52^ The dual Mo defects of Mo5 and Mo10 are found to facilitate the release of CO, with a corresponding decrease in net spin.

**Figure 5:**
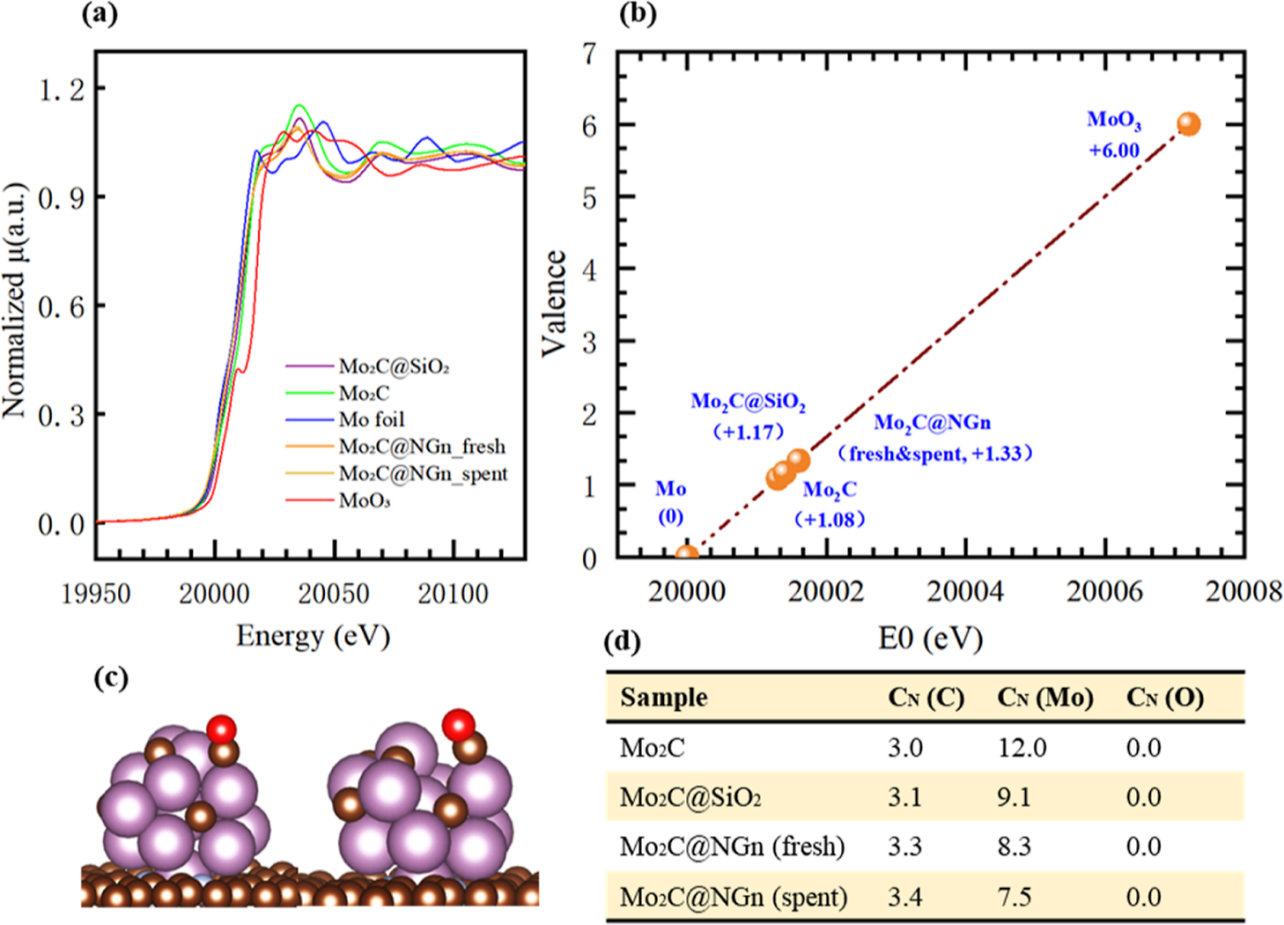
a) Mo K-edge XANES for the Mo-based catalysts. (b) Averaged valence of Mo in the specimens of Mo_2_C@SiO_2_, Mo_2_C@NGn (fresh), and Mo_2_C@NGn (spent) estimated with standard samples of Mo_2_C and metallic Mo. (c) Models of the intact and defected cluster of Mo_2_C supported on NGn. (d) Averaged coordination numbers of central Mo in the specimens of standard Mo_2_C, Mo_2_C@SiO_2_, Mo_2_C@NGn (fresh), and Mo_2_C@NGn (spent).

The effects of Mo defects on the CO_2_ adsorption were further investigated. Two different dual Mo defects (v5 and 10, v9 and v10) were compared with the intact Mo_2_C cluster, as shown in Figure 4b. It was found that all three clusters have obvious activation effects on CO_2_, wherein the bond angle of CO_2_ on the intact Mo_2_C sites was 118° and the C=O bond was 1.37 Å. On the cluster with dual-defects of Mo9 and Mo10, the C=O bond was more stretched to 1.40 Å. When it was on the cluster with dual-defects v5 and v10, the molecule was the most polarized with the smallest bond angle of 112°. This indicates the dual-defected Mo_2_C@NGn outperforms the intact cluster catalyst for CO_2_ activation as well.^22^ More interestingly, we observed that dissociation of CO_2_ on Mo9_v5 and 10 would give rise to the facile desorption of CO, while in other two circumstances, the CO molecule would stay binding with adjacent Mo atoms. The above results have clearly indicated that Mo defects create the original activity for the Mo_2_C@NGn cluster in the RWGS reaction. The above conclusion is further evidenced by experimental data. Figure 5 shows the results of X-ray absorption spectroscopy (XAS) for the catalyst samples of Mo_2_C@NGn (fresh), Mo_2_C@NGn (spent), and Mo_2_C@SiO_2_, as well as standard samples of Mo foil, MoO_3_, and Mo_2_C as references. XRD tests were implemented to the sample of Mo_2_C@NGn and Mo_2_C@SiO_2_ to confirm that the bulk species are entirely Mo_2_C, as shown in Figure S9. Figure 5a shows the X-ray absorption near-edge structure (XANES) spectra of the Mo K-edge for the investigated Mo-based catalysts. Figure 5b shows an average valence estimation for the Mo element in the catalysts and standard samples based on their edge positions. The valence of Mo in Mo_2_C@SiO_2_ was +1.17, close to that of Mo_2_C (standard sample), and the valence of Mo was +1.33 in both fresh Mo_2_C@NGn and spent Mo_2_C@NGn. The results imply a lower stoichiometric number of Mo molecules in the NGn-supported Mo_2_C catalysts.

We further analyzed the nearest coordination environment for all three samples in comparison with the Mo_2_C standard sample, based on the extended X-ray absorption fine structure (EXAFS) data. The detailed fitting paths and results are shown in Figure S10. Figure 5d shows the averaged coordinated number of adjacent elements for the Mo atom. As can be seen, for the Mo_2_C standard sample, one central Mo atom had three carbon atoms and 12 Mo in the first nearest neighbor, and when it was loaded on SiO_2_, the coordination number of C slightly increased, while the coordination number of Mo saw a slight decrease, implying the existence of Mo defects. When it was over the carbon support of NGn, the increase of C coordination number and the decrease of Mo coordination number became more obvious, indicating that rich Mo defects have been created on the Mo_2_C@NGn during carbonization and that the carbon support is effective to creating more defected sites, which is decisive to the catalytic activity toward the RWGS reaction.

### 2.3 Reaction Kinetics of Selective Hydrogenation over the Defected Mo-Based Catalysts

**Figure 6:**
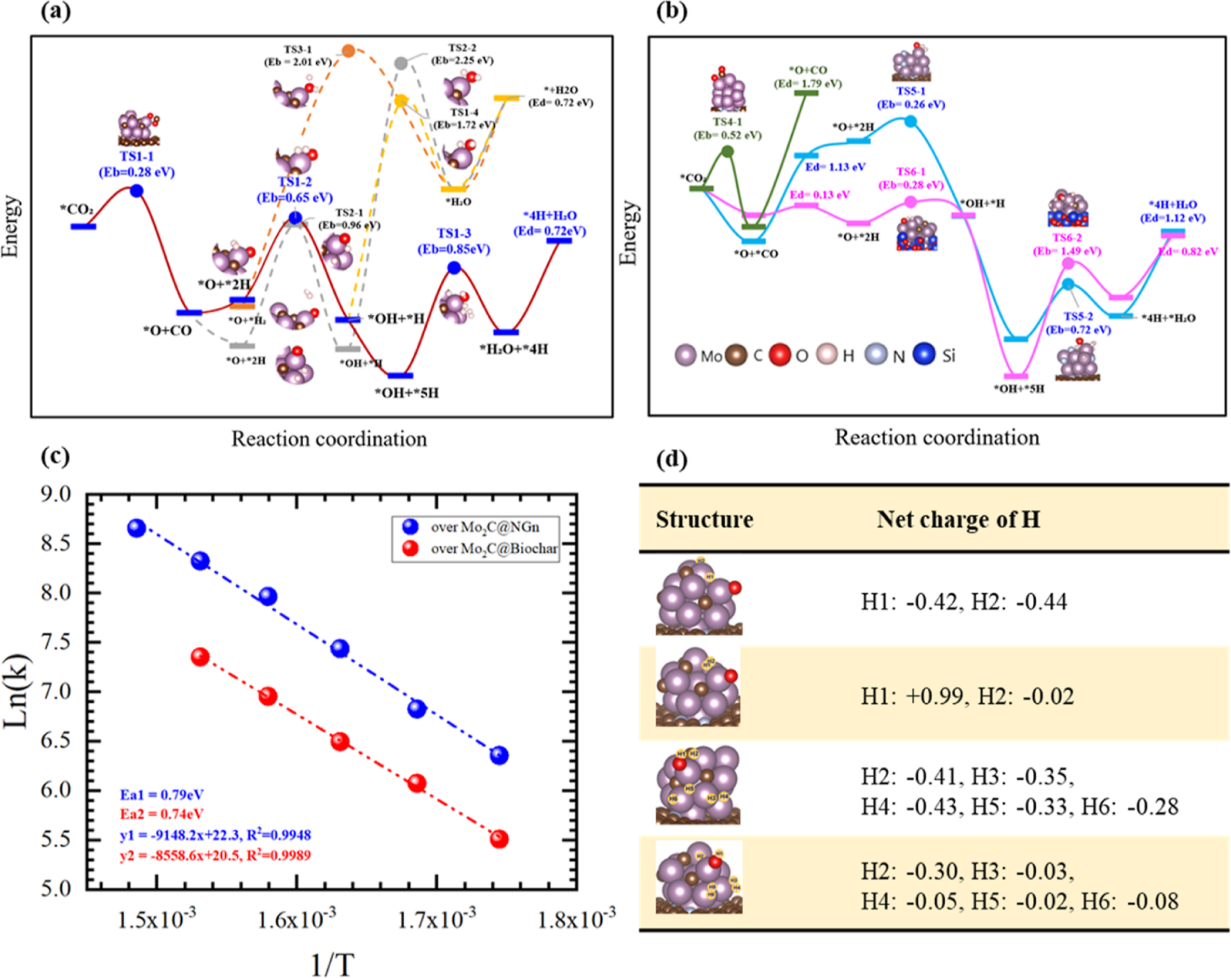
(a,b) Plausible catalytic reaction pathways of CO_2_ hydrogenation over Mo_2_C@NGn, Mo@NGn, Mo_2_N@NGn, and Mo_2_C@SiO_2_. (c) Apparent activation energy determined by experiments (mol_CO_2__/mol_H_2__ = 1:2, the peak conversion of CO_2_ was adjusted under 10%). (d) Atomic charges of the surface H atoms during the hydrogenation reaction.

Regarding the hydrogenation of CO_2_ over Mo_2_C_vMo@NGn, we considered the Mars–Van Krevelen (MVK) mechanism and the Eley–Rideal (ER) mechanism. Modeling results show that the reaction at the beginning could hardly proceed through the ER mechanism (Figure S11). Figure 6 demonstrates various hydrogenation pathways of CO_2_ and the corresponding potential energy barriers based on MVK mechanisms. We observed that CO_2_ experienced a small energy barrier of 0.28 eV before the direct dissociative desorption of CO. Three different pathways have been observed for hydrogenation to the surface *O, based on different configurations of adsorbed H_2_. The most favorable path had an energy barrier of 0.65 eV to produced *OH. The following step of hydrogenation with the only one surface *H needs to overcome energy barriers of at least 1.72 eV for both pathways. However, with additional hydrogen molecules coverage, the energy barrier was decreased by 0.87 eV to generate *H_2_O, which would further experience an endothermic desorption with the energy of 0.72 eV. In contract, one step hydrogenation of *O to produce *H_2_O under the ER mechanism had a much higher energy barrier of 2.01 eV. The hydrogenation pathways of CO_2_ over other catalysts of Mo_2_C_vMo@SiO_2_, Mo_vMo@NGn, and Mo_2_N_vMo@NGn were also modeled and are compared in Figure 6b. Over the defected Mo_2_C supported on SiO_2_, the dissociation of CO_2_ and hydrogenation to *O were facile, but hydrogenation to*OH had an energy barrier of 1.49 eV. Over the defected metallic Mo cluster supported on NGn, we found that the dissociation of CO_2_ was not difficult, while the CO desorption was the potential rate-determining step with an endothermic energy of 1.79 eV. The defected Mo_2_N supported on NGn gave rise to smaller energy barriers of 0.28 and 0.72 eV for the two steps hydrogenation, respectively, compared to those over Mo_2_C_vMo@NGn; however, the desorption energy of CO was 1.13 eV in the presence of *O.

The DFT modeling results confirm Mo_2_C_vMo@NGn to be the best catalyst candidate for the RWGS reaction, with the peak energy barrier of 0.85 eV for the most favorable pathway, and the result has been validated by the apparent activation energy of 0.79 eV determined by the experimental data, as shown in Figure 6c. The results in this study indicate that the peak energy for the RWGS reaction is 0.3 eV lower than that over the single-atom catalyst Mo1@NGn.^29^ Besides, we also carried out kinetic analysis for the experiments based on Mo_2_C@NBiochar, despite its slightly smaller turn over frequency (TOF) compared to Mo_2_C@NGn (Table S12). It demonstrates a lower activation energy in experiments, implying biochar as an alternative carbon support for large-scale applications instead of NGn.

In addition, the above modeling results also reveal that increasing hydrogen coverage would benefit the hydrogenation reaction, and this phenomenon was also observed in other hydrogenation circumstance^53^ and had been thoroughly investigated in our recent work;^29^ additional H_2_ adsorption is able to adjust the valent electrons of Mo atom and tends to neutralize the charge of surface *H atoms, promoting the hydrogenation. The conclusion was again verified by the hydrogenation in this study, where we found that only charge-neutral *H atoms were able to launch the hydrogenation, as shown in Figure S13. Interestingly, we noticed that Mo defects also contribute to the selective hydrogenation by neutralizing the *H charge. To clarify this, we carried out further comparative studies regarding the net charge of surface hydrogens over both intact Mo_2_C@NGn and Mo_2_C_vMo@NGn. Figure 6 reveals that the defected catalyst always leads to the charge neutrality of the surface *H. In contrast, over the intact cluster, the *H carried obvious negative charges in both circumstances, which would directly hinder the consequent hydrogenation reaction. We therefore conclude that Mo defects in Mo_2_C not only contribute to the CO_2_ activation and CO liberation but also benefit the following hydrogenations by proper charge adjustment of the surface hydrogen.

## 3 Conclusions and the Perspectives

In summary, we have demonstrated the outstanding performance of Mo_2_C@NGn in the low-temperature RWGS reaction. The catalyst achieved a CO yield rate of 6.3 g_CO_/g_cat_/h and >99% CO selectivity at 400 °C with good stability. Mechanism studies revealed that Mo_2_C@NGn outperformed other Mo-based catalysts by facilitating the dissociation of CO_2_ with nonbarrier liberation of CO, and the peak activation energy for the hydrogenation was around 0.8 eV, superior to the single-atom catalyst of Mo. We conclusively proved that Mo defects in Mo_2_C played a key role in driving the catalytic RWGS reaction. We observed that the atomic net spin was a potential descriptor for CO desorption based on a revealed linear correlation. The Mo defects facilitated product desorption by decreasing the magnetization of the adsorption site atom. On the other hand, the defects also neutralized the surface hydrogen charge, which was crucial for selective hydrogenation, in line with the function of increasing H coverage. Moreover, we have demonstrated that a combination of carbon support and carbonization process is an effective strategy for creating rich Mo defects to promote the selective hydrogenation of CO_2_ at low temperatures. Biochar, as an alternative support, gains much potential for large-scale applications, given its low cost and wide accessibility.

## 4 Methodology

### 4.1 Materials and Preparation of Catalysts

Materials of ammonium molybdate tetrahydrate [(NH4)_6_Mo_7_O_24_·4H_2_O], chromium(III) nitrate nonahydrate (CrN_3_O_9_·9H_2_O), ammonium tungstate [(NH_4_)10H_2_(W_2_O_7_)_6_], nitrogen-doped graphene (NGn), and SiO_2_ were supplied by Aladdin Reagents Co., Ltd. The NBiochar was prepared by lignin, urea, and NaHCO_3_ with the ratio 1/4/3 of each component; in each batch of preparation, 30 g of mixed feedstock was pyrolyzed in a graphite crucible at 700 °C for 2 h with a ramp of 3 °C/min in N_2_. The naturally cooled solid products were then washed by 1 M HNO_3_ solution and then with water several times until the pH of the solution was 7. The as-obtained Nbiochar was dried at 60 °C for 24 h before being used as the support in catalyst synthesis. The other batch of Nbiochar was prepared in the same process except the pyrolysis temperature of 800 °C. SiO_2_ was milled and sieved to 0.125–0.250 mm before being used as the support.

50% Mo_2_C@NGn catalysts were synthesized by wet impregnation with 50 mg of NGn with aqueous solutions of 92.1 mg of the metal precursor of (NH4)_6_Mo_7_O_24_·4H_2_O. The impregnation mixtures were stirred within a shaker for 24 h at room temperature (∼25 °C). Water was removed by evaporation at 80 °C, and the impregnated support was then dried at 60 °C for 24 h. The sample was then calcined in pure N_2_ for 2 h at 500 °C. The metal loading ratio was verified by the inductively coupled plasma (ICP) technique on the Thermo IRIS Intrepid II ICP–OES equipment. Specific surface area and porous information were investigated in the Brunauer–Emmett–Teller test. Relevant physical parameters are summarized in Table S14.

For carbonization before each experiment, the catalysts were pretreated in situ with 20 vol % CH_4_/H_2_ (total flow rate of 25 mL/min), with a ramp of 10 °C/min to 300 and 2 °C/min to 700 °C, and the temperature was stabilized at 700 °C for an additional 2 h. For reduction, the catalysts were pretreated in situ with 50 vol % H_2_/Ar (total flow rate of 20 mL/min) before each experiment, with a ramp of 10 °C/min to 700 °C, and the temperature was held at 700 °C for an additional 2 h. For nitrogenation, the catalysts were pretreated in situ with 50 vol % H_2_/N_2_ (total flow rate of 20 mL/min) before each experiment, with a ramp of 10 °C/min to 700 °C before the temperature was held at 700 °C for an additional 2 h. Catalysts with various metal loadings and metal species were prepared in an analogous procedure, except that the carbonization temperature for Cr_3_C_2_ was 850 °C. The as-obtained catalysts are denoted as *x*% M@NGn, where M is the active species and *x* is the loading ratio (*x* = 10, 20, or 50).

### 4.2 Structural Characterizations

In situ XRD patterns were recorded on a Bruker D8 Advance diffractometer equipped with an Anton Paar XRK-900 furnace operating at 40 kV with Cu Kα radiation (λ = 1.5406 Å) within the range of 20.0–50.0°, using a scanning rate of 0.85°/min. 30 mg of 50% Mo@NGn was exposed to a CH_4_/H_2_ flow (5 mL/min CH_4_ with 20 mL/min H_2_) for reduction. After the first XRD collection at room temperature, the temperature was sequentially increased to 300 °C with a ramp of 10 °C/min and then to 700 °C with a ramp of 2 °C/min, during which the XRD measurements were performed every 100 °C before 500 °C and every 20 °C afterward. The temperature was then held at 700 °C for another 6 h, and the XRD measurements were performed every 30 min. When the specimen was cooled to 500 °C, the XRD collection was then performed every 30 min in the reaction flow (5 mL/min of CO_2_, 10 mL/min of H_2_, and 5 mL/min of N_2_) for 3 h. The inlet gas was then switched to 10 mL/min H_2_ and 10 mL/min Ar for XRD measurements at constant 500 °C at the final 3 h. Ex situ XRD patterns were recorded on a RIGAKU SmartLab X-ray diffractometer with a scanning rate of 2°/min in the range of 20.0°–80.0°.

High-resolution transmission electron microscopy and EDS element mapping images were obtained with a Talos F200S field-emission high resolution TEM operating at 200 kV, and the information resolution was 0.12 nm. Specimens were prepared by ultrasonic dispersion of catalyst samples in ethanol before the suspension was dropped onto a copper grid. Images of the microstructures of the specimens were acquired. EDS elemental mapping was performed using SUPER-X to determine the existing elements on the sample surface.

XAS was performed at beamline 07 A of the Taiwan photon source (TPS) with the edge energy ranging from 5 to 22 keV. The data of standard samples were provided by TPS. Data processing of XANES and EXAFS was carried out using the Athena software package.

The CO temperature-programmed desorption (CO-TPD) experiments were performed on an Agilent 8860 instrument equipped with a thermal conductivity detector (TCD). Prior to the measurement, 10 mg of the catalyst precursor was loaded in a quartz tube, pretreated at 200 °C under Ar flow (20 mL/min) for 1 h to remove the adsorbed species on the catalyst surface, and then cooled to 30 °C in pure Ar flow. The catalysts were kept in a CO flow (20 mL/min) for 30 min to ensure saturated adsorption. Afterward, the adsorbed CO on catalysts was removed by pure Ar flow at 30 °C, and then, the CO-TPD process was carried out from 30 to 600 °C at a heating rate of 10 °C/min. The output signal was detected with a TCD. A similar TPD test was carried out from 80 to 600 °C. The difference of the amount for the desorbed CO in above two tests was regarded as weakly adsorbed CO and used for evaluation of the number of active sites on 50% Mo@NGn for the RWGS reaction.

### 4.3 Catalytic Performance Tests

CO_2_ hydrogenation experiments were carried out in a flow reactor under atmospheric pressure, in which the thermocouple was sleeved by a quartz tube to prevent the additional catalytic effects of the thermocouple on the reactions. In a typical batch, 10 mg of the catalyst precursor was loaded into a quartz tube with an inner diameter of 4 mm and held in place by quartz wool. Before each CO_2_ hydrogenation experiment, the catalyst was pretreated under the aforementioned conditions. The reactor was then cooled to 300 °C in the pretreatment flow, and the inlet flow was switched to the reactants (i.e., 5 mL/min CO_2_, 10 mL/min H_2_, and 5 mL/min N_2_) for CO_2_ hydrogenation. The temperature would remain constant at 300, 400, and 500 °C for reaction at each temperature in sequence with a ramp of 10 °C/min. The activation energy (*E*_a_) was determined according to the Arrhenius equation, based on the CO_2_ hydrogenation data between 300 and 400 °C with 20 °C intervals, and the GHSV was adjusted to keep a low conversion of no more than 10%. The gas chromatograph (Agilent 7890B) equipped with a flammable ionization detector (FID) and a TCD were used to analyze the concentration of gas products. The response factor of each reactant and product was calibrated using standard curve methods. The conversion of CO_2_ (*X*_CO_2__) is defined as
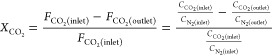
1

The selectivity of CO (*S*_CO_) is defined as
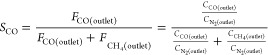
2where *F* is the flow rate of reactants or products (mol/min) and *C* is the concentration of reactants or products (%).

For each temperature test, an averaged value of 5 GC-FID/TCD data was used in this study. Exclusion of the reverse Boudouard reaction was confirmed by detection of the CO yield with an inactive catalyst, as shown in Table S16.

### 4.4 DFT Calculations

The first-principles density functional theory plus dispersion calculations were implemented in the Vienna Ab initio Simulation Package (VASP) with dispersion corrections by the D3 method of Grimme. The generalized gradient corrected approximation^54^ treated by the Perdew–Burke–Ernzerhof exchange–correlation potential was used to calculate the exchange–correlation energy.^55–57^ The Projector-Augmented-Wave (PAW) pseudopotential was employed as the scheme in the representation of reciprocal space for all the elements.^58,59^ The plane-wave cutoff energy was set to 450 eV for all the calculations. The Brillouin zone was sampled using a 2 × 2 × 1 and 4 × 4 × 1 Monkhorst–Pack *k*-point with a smearing of 0.1 eV for respective geometry optimization and static electron structures calculation. Independence tests of both cutoff energy and *k*-points were carried out, as shown in Figure S17. Spin polarization has been considered, and the self-consistent field tolerance was set to 10^–4^ eV/atom. All the modeling was performed with a convergence threshold of 0.03 eV/Å on maximum force. No symmetry constraint was used for any modeling. The computational method is believed to give high precision results and has been validated by experimental data in describing graphene-based materials in our recent work and Mo lattice.^29^ In this work, the model predicted the lattice constant of the Mo_2_C lattice to be *a* = 4.72 Å, *b* = 5.20 Å, and *c* = 6.02 Å, in agreement with the experimental data of *a* = 4.74 Å, *b* = 5.21 Å, and *c* = 6.03 Å,^60^ as shown in Table S18.

All the models were based on a three-layer *p*(4 × 4) supercell of four nitrogen-doped graphene (100) facet. A 15 Å vacuum region was created above the top layer of the graphite facet. MedeA 3.1.0 was used for model establishment and pseudopotential assembly for the calculation. Geometry optimization was implemented to each model before the energy was calculated. Energy of all of the geometries was calculated at 0 K in the DFT investigation with corrections of zero point energy based on frequency analysis. Bader charge was calculated for atomic electron analysis.^61,62^ The adsorption energy (*E*_ad_) and desorption energy (*E*_des_) are determined by eqs 3 and 4, where *E*_catalyst_, *E*_adsorbate_, and *E*_adsorbate/catalyst_ are the total energies of clean catalyst, free adsorbate molecule, and catalyst with adsorbed molecule, respectively. The transition state (TS) was completely determined by the algorithm of climbing image nudged elastic band combining with the dimer method and confirmed by sole imaginary frequencies (Figure S15). The energy barrier of a reaction (*E*_barrier_) was determined by the difference between the energies of the TS and reactant, as shown in eq 5, where the *E*_transition_ state and reactant are the total energies of the TS and reactant of a reaction, respectively.
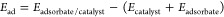
3
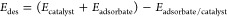
4
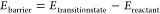
5

The electron density difference was determined by eq 6.
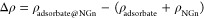
6where ρ_adsorbate@NGn_ is the electron density of the whole adsorbate + adsorbent system, and ρ_adsorbate_ and ρ_NGn_ are the unperturbed electron densities of the adsorbate and the adsorbent structure, respectively.

### 4.5 AIMD Modeling

The AIMD modeling was implemented to confirm the stability of the catalyst unit, within the canonical (*NVT*) ensemble in VASP 5.4.4, using the model established in this work. The time step was 0.5 fs, and the testing temperature was 1000 K.

## Supporting Information Available

Details of the catalytic behavior of reported catalysts in the literature and other mentioned catalysts in the article, cluster, and support interactions; Fukui analysis; bond strength analysis and electronic spin analysis; electronic spin of the clusters; EXAFS fittings; TOF of reaction; charge analysis of surface *H atoms; physical properties of the catalysts; TS frequencies; reaction test with inert catalyst; independence test of cutoff energy and *k*-points; and the validation of the computational method (PDF)

## References

[ref1] Dokania A.; Ould-Chikh S.; Ramirez A.; Cerrillo J. L.; Aguilar A.; Russkikh A.; Alkhalaf A.; Hita I.; Bavykina A.; Shterk G.; Wehbe N.; Prat A.; Lahera E.; Castaño P.; Fonda E.; Hazemann J. L.; Gascon J. Designing a Multifunctional Catalyst for the Direct Production of Gasoline-Range Isoparaffins from CO2. JACS Au 2021, 1 (11), 1961–1974. 10.1021/jacsau.1c00317. 34841412 PMC8611669

[ref2] Paykani A.; Chehrmonavari H.; Tsolakis A.; Alger T.; Northrop W. F.; Reitz R. D. Synthesis Gas as a Fuel for Internal Combustion Engines in Transportation. Prog. Energy Combust. Sci. 2022, 90 (February), 100995 10.1016/j.pecs.2022.100995.

[ref3] Du P.; Qi R.; Zhang Y.; Gu Q.; Xu X.; Tan Y.; Liu X.; Wang A.; Zhu B.; Yang B.; Zhang T. Single-Atom-Driven Dynamic Carburization over Pd1–FeOx Catalyst Boosting CO2 Conversion. Chem 2022, 8 (12), 3252–3262. 10.1016/j.chempr.2022.08.012.

[ref4] Zhang R.; Wang X.; Wang K.; Wang H.; Liu L.; Wu X.; Geng B.; Chu X.; Song S.; Zhang H. Synergism of Ultrasmall Pt Clusters and Basic La 2 O 2 CO 3 Supports Boosts the Reverse Water Gas Reaction Efficiency. Adv. Energy Mater. 2023, 13, 2203806 10.1002/aenm.202203806.

[ref5] Wang L.; Zhang L.; Zhang L.; Yun Y.; Wang K.; Yu B.; Zhao X.; Yang F. Direct Environmental TEM Observation of Silicon Diffusion-Induced Strong Metal-Silica Interaction for Boosting CO2 Hydrogenation. Nano Res. 2022, 16, 2209–2217. 10.1007/s12274-022-4991-1.

[ref6] Ziemba M.; Weyel J.; Hess C. Elucidating the Mechanism of the Reverse Water–Gas Shift Reaction over Au/CeO2 Catalysts Using Operando and Transient Spectroscopies. Appl. Catal., B 2022, 301 (October 2021), 120825 10.1016/j.apcatb.2021.120825.

[ref7] Chen L.; Unocic R. R.; Hoffman A. S.; Hong J.; Braga A. H.; Bao Z.; Bare S. R.; Szanyi J. Unlocking the Catalytic Potential of TiO2-Supported Pt Single Atoms for the Reverse Water-Gas Shift Reaction by Altering Their Chemical Environment. JACS Au 2021, 1 (7), 977–986. 10.1021/jacsau.1c00111. 34467344 PMC8395703

[ref8] Kim G.; Shin S.; Choi Y.; Kim J.; Kim G.; Kim K.-J.; Lee H. Gas-Permeable Iron-Doped Ceria Shell on Rh Nanoparticles with High Activity and Durability. JACS Au 2022, 2 (5), 1115–1122. 10.1021/jacsau.2c00035. 35647595 PMC9131474

[ref9] Lin S.; Wang Q.; Li M.; Hao Z.; Pan Y.; Han X.; Chang X.; Huang S.; Li Z.; Ma X. Ni–Zn Dual Sites Switch the CO 2 Hydrogenation Selectivity via Tuning of the d-Band Center. ACS Catal. 2022, 12 (6), 3346–3356. 10.1021/acscatal.1c05582.

[ref10] Sinehbaghizadeh S.; Saptoro A.; Mohammadi A. H. CO2 hydrate properties and applications: A state of the art. Nat. Commun. 2022, 93 (1), 101026 10.1016/j.pecs.2022.101026.

[ref11] Wei A.; Zhang R.; Qin Y.; Wang H.; Zhu X.; Ge Q. Theoretical Insight into Tuning CO_2_ Methanation and Reverse Water Gas Shift Reactions on MoO_*x*_-Modified Ni Catalysts. J. Phys. Chem. C 2022, 126 (42), 18078–18089. 10.1021/acs.jpcc.2c03216.

[ref12] Li W.; Nie X.; Yang H.; Wang X.; Polo-Garzon F.; Wu Z.; Zhu J.; Wang J.; Liu Y.; Shi C.; Song C.; Guo X. Crystallographic Dependence of CO2 Hydrogenation Pathways over HCP-Co and FCC-Co Catalysts. Appl. Catal., B 2022, 315 (May), 121529 10.1016/j.apcatb.2022.121529.

[ref13] Xing S.; Turner S.; Fu D.; van Vreeswijk S.; Liu Y.; Xiao J.; Oord R.; Sann J.; Weckhuysen B. M. Silicalite-1 Layer Secures the Bifunctional Nature of a CO 2 Hydrogenation Catalyst. JACS Au 2023, 3 (4), 1029–1038. 10.1021/jacsau.2c00621. 37124291 PMC10131208

[ref14] Li K.; Li X.; Li L.; Chang X.; Wu S.; Yang C.; Song X.; Zhao Z. J.; Gong J. Nature of Catalytic Behavior of Cobalt Oxides for CO2 Hydrogenation. JACS Au 2023, 3 (2), 508–515. 10.1021/jacsau.2c00632. 36873681 PMC9975827

[ref15] Li Y.; Zhao Z.; Lu W.; Zhu H.; Sun F.; Mei B.; Jiang Z.; Lyu Y.; Chen X.; Guo L.; Wu T.; Ma X.; Meng Y.; Ding Y. Single-Atom Co-N-C Catalysts for High-Efficiency Reverse Water-Gas Shift Reaction. Appl. Catal., B 2023, 324 (November 2022), 122298 10.1016/j.apcatb.2022.122298.

[ref16] Cao F.; Xiao Y.; Zhang Z.; Li J.; Xia Z.; Hu X.; Ma Y.; Qu Y. Influence of Oxygen Vacancies of CeO2 on Reverse Water Gas Shift Reaction. J. Catal. 2022, 414, 25–32. 10.1016/j.jcat.2022.08.021.

[ref17] Pajares A.; Prats H.; Romero A.; Viñes F.; de la Piscina P. R.; Sayós R.; Homs N.; Illas F. Critical Effect of Carbon Vacancies on the Reverse Water Gas Shift Reaction over Vanadium Carbide Catalysts. Appl. Catal., B 2020, 267 (February), 118719 10.1016/j.apcatb.2020.118719.

[ref18] Zhang Q.; Pastor-Pérez L.; Wang Q.; Ramirez Reina T. Conversion of CO2 to Added Value Products via RWGS Using Fe-Promoted Catalysts: Carbide, Metallic Fe or a Mixture?. J. Energy Chem. 2022, 66, 635–646. 10.1016/j.jechem.2021.09.015.

[ref19] Pahija E.; Panaritis C.; Gusarov S.; Shadbahr J.; Bensebaa F.; Patience G.; Boffito D. C. Experimental and Computational Synergistic Design of Cu and Fe Catalysts for the Reverse Water–Gas Shift: A Review. ACS Catal. 2022, 12 (12), 6887–6905. 10.1021/acscatal.2c01099.

[ref20] Li Y.; Fang Z.; Zhou H.; Li Y.; Wang B.; Huang S.; Lin W.; Chen W. K.; Zhang Y. Theoretical Insights into Synergistic Effects at Cu/TiC Interfaces for Promoting CO2Activation. ACS Omega 2021, 6 (41), 27259–27270. 10.1021/acsomega.1c04040. 34693146 PMC8529663

[ref21] Morales-Salvador R.; Gouveia J. D.; Morales-García Á.; Viñes F.; Gomes J. R. B.; Illas F. Carbon Capture and Usage by MXenes. ACS Catal. 2021, 11 (17), 11248–11255. 10.1021/acscatal.1c02663.

[ref22] Jimenez-Orozco C.; Figueras M.; Flórez E.; Viñes F.; Rodriguez J. A.; Illas F. Effect of Nanostructuring on the Interaction of CO 2 with Molybdenum Carbide Nanoparticles. Phys. Chem. Chem. Phys. 2022, 24 (27), 16556–16565. 10.1039/D2CP01143C. 35770743

[ref23] Figueras M.; Gutiérrez R. A.; Viñes F.; Ramírez P. J.; Rodriguez J. A.; Illas F. Supported Molybdenum Carbide Nanoparticles as Hot Hydrogen Reservoirs for Catalytic Applications. J. Phys. Chem. Lett. 2020, 11 (19), 8437–8441. 10.1021/acs.jpclett.0c02608. 32960609

[ref24] Zhang Q.; Bown M.; Pastor-Pérez L.; Duyar M. S.; Reina T. R. CO 2 Conversion via Reverse Water Gas Shift Reaction Using Fully Selective Mo–P Multicomponent Catalysts. Ind. Eng. Chem. Res. 2022, 61 (34), 12857–12865. 10.1021/acs.iecr.2c00305. 36065445 PMC9437872

[ref25] Zhou H.; Chen Z.; Kountoupi E.; Tsoukalou A.; Abdala P. M.; Florian P.; Fedorov A.; Müller C. R. Two-Dimensional Molybdenum Carbide 2D-Mo2C as a Superior Catalyst for CO2 Hydrogenation. Nat. Commun. 2021, 12 (1), 5510 10.1038/s41467-021-25784-0. 34535647 PMC8448824

[ref26] Wu Y.; Xie Z.; Gao X.; Zhou X.; Xu Y.; Fan S.; Yao S.; Li X.; Lin L. The Highly Selective Catalytic Hydrogenation of CO2 to CO over Transition Metal Nitrides. Chin. J. Chem. Eng. 2022, 43, 248–254. 10.1016/j.cjche.2021.12.022.

[ref27] Liu H.-X.; Li J.-Y.; Qin X.; Ma C.; Wang W.-W.; Xu K.; Yan H.; Xiao D.; Jia C.-J.; Fu Q.; Ma D. Ptn–Ov Synergistic Sites on MoOx/γ-Mo2N Heterostructure for Low-Temperature Reverse Water–Gas Shift Reaction. Nat. Commun. 2022, 13 (1), 5800 10.1038/s41467-022-33308-7. 36192383 PMC9530113

[ref28] Feng K.; Tian J.; Zhang J.; Li Z.; Chen Y.; Luo K. H.; Yang B.; Yan B. Dual Functionalized Interstitial N Atoms in Co 3 Mo 3 N Enabling CO 2 Activation. ACS Catal. 2022, 12 (8), 4696–4706. 10.1021/acscatal.2c00583.

[ref29] Zhang J.; Yang B.; Luo K. H. Unveiling the Mechanism of Controllable CO2 Hydrogenation by Group VIB Metal Single Atom Anchored on N-Doped Graphite: A Density Functional Theory Study. Int. J. Hydrogen Energy 2022, 47 (97), 40972–40985. 10.1016/j.ijhydene.2022.09.170.

[ref30] Jiang Y.; Sung Y.; Choi C.; Joo Bang G.; Hong S.; Tan X.; Wu T. S.; Soo Y. L.; Xiong P.; Meng-Jung Li M.; Hao L.; Jung Y.; Sun Z. Single-Atom Molybdenum-N3 Sites for Selective Hydrogenation of CO2 to CO. Angew. Chem., Int. Ed. 2022, 61 (37), e202203836 10.1002/anie.202203836. 35852815

[ref31] Kunisada Y.; Sakaguchi N. Chemical Modification of Graphene for Atomic-Scale Catalyst Supports. Nano Express 2022, 3, 042001 10.1088/2632-959X/aca41f.

[ref32] Nie Y.; Deng J.; Chen S.; Wei Z. Promoting Stability and Activity of PtNi/C for Oxygen Reduction Reaction via Polyaniline-Confined Space Annealing Strategy. Int. J. Hydrogen Energy 2019, 44 (12), 5921–5928. 10.1016/j.ijhydene.2019.01.125.

[ref33] Mao S.; Wang C.; Wang Y. The Chemical Nature of N Doping on N Doped Carbon Supported Noble Metal Catalysts. J. Catal. 2019, 375, 456–465. 10.1016/j.jcat.2019.06.039.

[ref34] Nityashree N.; Price C. A. H.; Pastor-Perez L.; Manohara G. V.; Garcia S.; Maroto-Valer M. M.; Reina T. R. Carbon Stabilised Saponite Supported Transition Metal-Alloy Catalysts for Chemical CO2 Utilisation via Reverse Water-Gas Shift Reaction. Appl. Catal., B 2020, 261, 118241 10.1016/j.apcatb.2019.118241.

[ref35] Rudy E.; Windisch S.; Stosick A. J.; Hoffman J. R. The Constitution of Binary Molybdenum-Carbon Alloys. Trans. Metall. Soc. AIME 1967, 239, 1247–1267.

[ref36] Lv C.; Yang Q.; Xu S.; Yuan L.; Huang Z.; Ren Z.; Luo J.; Wang S.; Zhang C. The in Situ Removal of Surface Molybdenum Oxide for Making Binder-Free Porous Mo 1.98 C 1.02 Film a More Efficient Electrocatalyst for Alkaline Rather than Acidic Hydrogen Production. Sustainable Energy Fuels 2021, 5 (13), 3373–3381. 10.1039/D1SE00452B.

[ref37] Lv C.; Lou P.; Shi C.; Wang R.; Fu Y.; Gao L.; Wang S.; Li Y.; Zhang C. Efficient Hydrogen Production via Sunlight-Driven Thermal Formic Acid Decomposition over a Porous Film of Molybdenum Carbide. J. Mater. Chem. A 2021, 9 (39), 22481–22488. 10.1039/D1TA06059G.

[ref38] Ettmayer P. The System Molybdenum-Nitrogen. Monatsh. Chem. 1970, 101 (1), 127–140. 10.1007/BF00907533.

[ref39] Naghdi S.; Rhee K. Y.; Kim M. T.; Jaleh B.; Park S. J. Atmospheric Chemical Vapor Deposition of Graphene on Molybdenum Foil at Different Growth Temperatures. Carbon Lett. 2016, 18, 37–42. 10.5714/CL.2016.18.037.

[ref40] Ji J.; Aleisa R. M.; Duan H.; Zhang J.; Yin Y.; Xing M. Metallic Active Sites on MoO2(110) Surface to Catalyze Advanced Oxidation Processes for Efficient Pollutant Removal. iScience 2020, 23 (2), 100861 10.1016/j.isci.2020.100861. 32058972 PMC7011042

[ref41] Pan W.; Tian R.; Jin H.; Guo Y.; Zhang L.; Wu X.; Zhang L.; Han Z.; Liu G.; Li J.; Rao G.; Wang H.; Chu W. Structure, Optical, and Catalytic Properties of Novel Hexagonal Metastable h -MoO 3 Nano- and Microrods Synthesized with Modified Liquid-Phase Processes. Chem. Mater. 2010, 22 (22), 6202–6208. 10.1021/cm102703s.

[ref42] Liu X.; Xing C.; Yang F.; Liu Z.; Wang Y.; Dong T.; Zhao L.; Liu H.; Zhou W. Strong Interaction over Ru/Defects-Rich Aluminium Oxide Boosts Photothermal CO 2 Methanation via Microchannel Flow-Type System. Adv. Energy Mater. 2022, 12 (31), 2201009 10.1002/aenm.202201009.

[ref43] Dewangan N.; Ashok J.; Sethia M.; Das S.; Pati S.; Kus H.; Kawi S. Cobalt-Based Catalyst Supported on Different Morphologies of Alumina for Non-oxidative Propane Dehydrogenation: Effect of Metal Support Interaction and Lewis Acidic Sites. ChemCatChem 2019, 11 (19), 4923–4934. 10.1002/cctc.201900924.

[ref44] Chen J.; Wang H.; Wang Z.; Mao S.; Yu J.; Wang Y.; Wang Y. Redispersion of Mo-Based Catalysts and the Rational Design of Super Small-Sized Metallic Mo Species. ACS Catal. 2019, 9 (6), 5302–5307. 10.1021/acscatal.8b04634.

[ref45] Marquart W.; Raseale S.; Prieto G.; Zimina A.; Sarma B. B.; Grunwaldt J.-D.; Claeys M.; Fischer N. CO 2 Reduction over Mo 2 C-Based Catalysts. ACS Catal. 2021, 11 (3), 1624–1639. 10.1021/acscatal.0c05019.

[ref46] Nan B.; Fu Q.; Yu J.; Shu M.; Zhou L.-L.; Li J.; Wang W.-W.; Jia C.-J.; Ma C.; Chen J.-X.; Li L.; Si R. Unique Structure of Active Platinum-Bismuth Site for Oxidation of Carbon Monoxide. Nat. Commun. 2021, 12 (1), 3342 10.1038/s41467-021-23696-7. 34099668 PMC8184822

[ref47] Yang Y.; Tan M.; Garcia A.; Zhang Z.; Lin J.; Wan S.; McEwen J.-S.; Wang S.; Wang Y. Controlling the Oxidation State of Fe-Based Catalysts through Nitrogen Doping toward the Hydrodeoxygenation of m -Cresol. ACS Catal. 2020, 10 (14), 7884–7893. 10.1021/acscatal.0c00626.

[ref48] Liu X.; Kunkel C.; Ramírez de la Piscina P.; Homs N.; Viñes F.; Illas F. Effective and Highly Selective CO Generation from CO_2_ Using a Polycrystalline α-Mo_2_C Catalyst. ACS Catal. 2017, 7 (7), 4323–4335. 10.1021/acscatal.7b00735.

[ref49] Li J.; Liu J.; Yang B. Insights into the Adsorption/Desorption of CO2 and CO on Single-Atom Fe-Nitrogen-Graphene Catalyst under Electrochemical Environment. J. Energy Chem. 2021, 53, 20–25. 10.1016/j.jechem.2020.04.016.

[ref50] Ram S.; Lee S. C.; Bhattacharjee S. Adsorption Energy Scaling Relation on Bimetallic Magnetic Surfaces: Role of Surface Magnetic Moments. Phys. Chem. Chem. Phys. 2020, 22 (32), 17960–17968. 10.1039/D0CP01382J. 32747888

[ref51] Wu P.; Huang M. Investigation of Adsorption Behaviors, and Electronic and Magnetic Properties for Small Gas Molecules Adsorbed on Pt-Doped Arsenene by Density Functional Calculations. RSC Adv. 2023, 13 (6), 3807–3817. 10.1039/D2RA08028A. 36756604 PMC9890969

[ref52] Dang Q.; Tang S.; Liu T.; Li X.; Wang X.; Zhong W.; Luo Y.; Jiang J. Regulating Electronic Spin Moments of Single-Atom Catalyst Sites via Single-Atom Promoter Tuning on S-Vacancy MoS2for Efficient Nitrogen Fixation. J. Phys. Chem. Lett. 2021, 12 (34), 8355–8362. 10.1021/acs.jpclett.1c02432. 34432475

[ref53] Figueras M.; Gutiérrez R. A.; Viñes F.; Ramírez P. J.; Rodriguez J. A.; Illas F. Supported Molybdenum Carbide Nanoparticles as an Excellent Catalyst for CO 2 Hydrogenation. ACS Catal. 2021, 11 (15), 9679–9687. 10.1021/acscatal.1c01738.

[ref54] Perdew J. P.; Burke K.; Ernzerhof M. Generalized Gradient Approximation Made Simple. Phys. Rev. Lett. 1996, 77 (18), 3865–3868. 10.1103/PhysRevLett.77.3865. 10062328

[ref55] Grimme S. Semiempirical GGA-Type Density Functional Constructed with a Long-Range Dispersion Correction. J. Comput. Chem. 2006, 27 (15), 1787–1799. 10.1002/jcc.20495. 16955487

[ref56] Lebedeva I. V.; Lebedev A. V.; Popov A. M.; Knizhnik A. A. Comparison of Performance of van Der Waals-Corrected Exchange-Correlation Functionals for Interlayer Interaction in Graphene and Hexagonal Boron Nitride. Comput. Mater. Sci. 2017, 128, 45–58. 10.1016/j.commatsci.2016.11.011.

[ref57] Politi J. R. D. S.; Viñes F.; Rodriguez J. A.; Illas F. Atomic and Electronic Structure of Molybdenum Carbide Phases: Bulk and Low Miller-Index Surfaces. Phys. Chem. Chem. Phys. 2013, 15 (30), 12617 10.1039/c3cp51389k. 23787949

[ref58] Lejaeghere K.; Van Speybroeck V.; Van Oost G.; Cottenier S. Error Estimates for Solid-State Density-Functional Theory Predictions: An Overview by Means of the Ground-State Elemental Crystals. Crit. Rev. Solid State Mater. Sci. 2014, 39 (1), 1–24. 10.1080/10408436.2013.772503.

[ref59] Pickard C. J.; Winkler B.; Chen R. K.; Payne M. C.; Lee M. H.; Lin J. S.; White J. A.; Milman V.; Vanderbilt D. Structural Properties of Lanthanide and Actinide Compounds within the Plane Wave Pseudopotential Approach. Phys. Rev. Lett. 2000, 85 (24), 5122–5125. 10.1103/PhysRevLett.85.5122. 11102201

[ref60] Epicier T.; Dubois J.; Esnouf C.; Fantozzi G.; Convert P. Neutron Powder Diffraction Studies of Transition Metal Hemicarbides M2C1–—II. In Situ High Temperature Study on W2C1– and Mo2C1. Acta Metall. 1988, 36 (8), 1903–1921. 10.1016/0001-6160(88)90293-3.

[ref61] Tang W.; Sanville E.; Henkelman G. A Grid-Based Bader Analysis Algorithm without Lattice Bias. J. Phys.: Condens. Matter 2009, 21 (8), 084204 10.1088/0953-8984/21/8/084204. 21817356

[ref62] Henkelman G.; Arnaldsson A.; Jónsson H. A Fast and Robust Algorithm for Bader Decomposition of Charge Density. Comput. Mater. Sci. 2006, 36 (3), 354–360. 10.1016/j.commatsci.2005.04.010.

