# Supplementary material for: Defect-Driven Efficient Selective CO2 Hydrogenation with Mo-Based Clusters
Source: JACS Au. 2023 Sep 15;3(10):2736–48. doi: 10.1021/jacsau.3c00206 (PMC10598559; doi:10.1021/jacsau.3c00206)
Supplement: Supplementary file 1 — au3c00206_si_001.pdf [file au3c00206_si_001.pdf]

# Defect-Driven Efficient Selective $CO_2$ Hydrogenation with Mo-Based Clusters

Jiajun Zhang[a,b], Kai Feng[c], Zhengwen Li[c], Bin Yang[b,*], Binhang Yan[c,*], Kai Hong Luo[b,d*]

[a] *National Engineering Research Center of Green Recycling for Strategic Metal Resources, Institute of Process Engineering, Chinese Academy of Sciences, Beijing 100190, China*

[b] *Center for Combustion Energy, Key Laboratory for Thermal Science and Power Engineering of Ministry of Education, International Joint Laboratory on Low Carbon Clean Energy Innovation, Tsinghua University, Beijing 100084, China*

[c] *Department of Chemical Engineering, Tsinghua University, Beijing 100084, China*

[d] *Department of Mechanical Engineering, University College London, Torrington Place, London WC1E 7JE, UK*

[*] Corresponding author: B.Yang., byang@tsinghua.edu.cn
[*] Corresponding author: B.Yan., binhangyan@tsinghua.edu.cn
[*] Corresponding author: K.H.L., k.luo@ucl.ac.uk

# Supporting Information

## Table of contents:

Table S1.Summary of reported catalysts for RWGS reaction ........................................... 4

Fig. S2. Particle size distribution of **a,** Fresh Mo@NGn-$CH_4$ and **b,** $Mo_2C$@$SiO_2$-$CH_4$......... 5

Fig. S3. (a) The optimized structure of $Mo_2C$@NGn, (b) final structure after 7ps AIMD modelling, differential electron density of (c) $Mo_2C$@NGn and (d) $Mo_2C$@$SiO_2$ shown with an electronic density isosurface of 0.015e/Å$^3$, where yellow colour denotes electron enrichment and cyan colour denotes electron loss. ......................................................................... 6

Fig. S4. The catalytic activity of Mo based catalysts pretreated under nitrogenation and reduction. The effects of (a) Loading ratio of Mo (b) metal species (c) preparation methods (d) calcination temperatures for NBiochar support under RWGS reaction conditions (GHSV = 140000ml/g/h, $mol_{CO2}$:$mol_{H2}$=1:2). .................................................................... 7

Fig. S5. Fukui analysis of an intact cluster of $Mo_2C$ supported on NGn. ............................. 8

Fig. S6. Fukui analysis of the dual-defected $Mo_2C$_V5&10@NGn. ................................... 9

Fig. S7. Independence of CO desorption energy and Mo-CO bond strength in a variety of $Mo_2C$ catalytic system. ........................................................................................ 10

Fig. S8. (a) Electronic spin of $Mo_2C$_V5&10@NGn and $Mo_2C$_V5&10@$SiO_2$ before and after CO adsorption. (b) The corresponding pDOS for Mo9 and C (in CO) in the two catalytic systems. ............................................................................................................ 11

Fig. S9. XRD patterns of the Mo based catalysts over NGn and $SiO_2$ respectively ............. 12

Fig. S10. The EXAFS results and curve fittings for Mo based species. (a) Mo K-edge EXAFS (points) and the curvefit (line) for $Mo_2C$@NGn_fresh, shown in k3-weighted k-space and R space. R-factor = 0.0180. (b) Mo K-edge EXAFS (points) and the curvefit (line) for $Mo_2C$@NGn_spent, shown in k3-weighted k-space and R space. R-factor = 0.0288. (c) Mo K-edge EXAFS (points) and the curvefit (line) for $Mo_2C$, shown in k3-weighted k-space and R space. R-factor = 0.0183. (d) Mo K-edge EXAFS (points) and the curvefit (line) for $Mo_2C$@$SiO_2$, shown in k3-weighted k-space and R space. R-factor = 0.0209. (e) Mo K-edge EXAFS (points) and the curvefit (line) for $MoO_3$, shown in k3-weighted k-space and R space. R-factor = 0.0258. The data are k3-weighted and not phase corrected. ............................. 14

Fig. S11. The plausible catalytic reaction in the ER mechanism ....................................... 14

Table S12. TOF of the $Mo_2C$ cluster supported on NGn and Nbiochar (GHSV = 930000ml/g/h, $mol_{CO2}$:$mol_{H2}$=1:2) ................................................................................ 15

Fig. S13. Charge analysis of surface *H atoms on $Mo_2N$_vMo@NGn and $Mo_2C$_vMo@$SiO_2$. ........................................................................................................................ 15

Table S14. Physical properties of the sample 50%Mo@NGn. .......................................... 16

Table S15. The imaginary frequency of the transition state structures for each elementary reaction shown in Fig. 7 (a) and (b) ............................................................................ 16

Table S16. Reaction tests over 1%W@NGn in comparison with Mo based catalyst ........... 17

Fig. S17. Independence tests for (a) cut-off energy and (b) k-points respectively ............... 17

Table S18. Validation of the present computational method............................................. 18

Table S1.Summary of reported catalysts for RWGS reaction

| Catalyst | Temp | GHSV ($ml/g_{cat}/h$) | $H_2/CO_2$ | Conversion | Conversion rate ($mmol/g_{cat}/h$) | CO selectivity | Ref |
|---|---|---|---|---|---|---|---|
| 100%$Mo_2C$ (porous) | 300°C | 36000 | 2:1 | 8.7% | 42.6 | 93.5% | [1] |
| 5%Co-N-C | 300°C | - | - | - | ~3.2 | ~80.0% | [2] |
| 0.05%$Pd_1$@$FeO_x$ | 300°C | - | - | - | 42.0 | 98.0% | [3] |
| 10%Cu-10%ZnO@SBA-15 | 300°C | 15000 | 3:1 | ~10.0% | 13.4 | ~99.9% | [4] |
| 0.16%Pt@$La_2O_2CO_3$ | 300°C | - | - | - | 22.0 | 100.0% | [5] |
| 13.9%Ni/13.5%Cu@Saponite | 500°C | 15000 | 4:1 | 53.0% | 58.4* | 89.0% | [6] |
| 15%Fe@$Al_2O_3$ | 500°C | 12500 | 4:1 | 40.0% | 40.8 | 92% | [7] |
| 1.7%Pt-1.5%Co/$CeO_2$ | 300°C | 36000 | 2:1 | 6.6% | 32.3 | 82.0% | [1,8] |
| 2%K-20%$Mo_2C$@γ-$Al_2O_3$ | 300°C | 3024 | 3:1 | 18.1% | 6.1 | 95.9% | [9] |
| 8.4wt%Cu/11at%$MnO_X$@C | 260°C | ~3479 | 3:1 | 20.0% | 7.1 | 87.0% | [10] |
| 5%Ni-8%Mo@$SiO_2$ | 400°C | 50000 | 4:1 | ~30.0% | 61.2 | 95.0% | [11**] |
| 100%2D-$Mo_2C$ | 230°C | ~120000 | 3:1 | - | 26.8 | 65% | [12] |
| 2%Ni@$Co_3O_4$ | 300°C | 200000 | 1:1 | 4.0% | 35.7 | 95% | [13] |
| $Mo_2CT_x$*** | 230°C | ~120000 | 3:1 | - | 5.6 | 71% | [12] |
| 100%β-Mo2C | 230°C | ~120000 | 3:1 | - | 3.3 | 67% | [12] |
| 100%α-Mo2C | 325°C | - | 1:1 | ~6.5% | 5.2 | 97.8% | [14] |
| 5%Co/γ-$Al_2O_3$ | 300°C | - | 5:1 | 15.0% | 60.8 | 100% | [15] |
| 5%Co/(Si)γ-$Al_2O_3$ | 300°C | - | 5:1 | 15.0% | 60.8 | 83% | [15] |
| 0.5%Pt/$CeO_2$@$SiO_2$ | 350°C | 66000 | 3:1 | 9% | 48.2 | 99% | [16] |
| WC/γ-$Al_2O_3$ | 350°C | 9000 | 3:1 | ~17% | 14.3 | ~91.5% | [17] |
| 1%Cu-$Mo_2C$ | 400°C | 12000 | 4:1 | ~39% | 41.8 | ~86% | [18] |
| 50%$Mo_2C$@NGn | 300°C | 140000 | 2:1 | 4.88% | 69.4 | 98.0% | This work |

* In the case that reactants were balanced by 10% $N_2$. ** Stable performance of the catalyst was adopted. *** $T_x$ denotes surface oxo, hydroxy, and/or fluoro groups

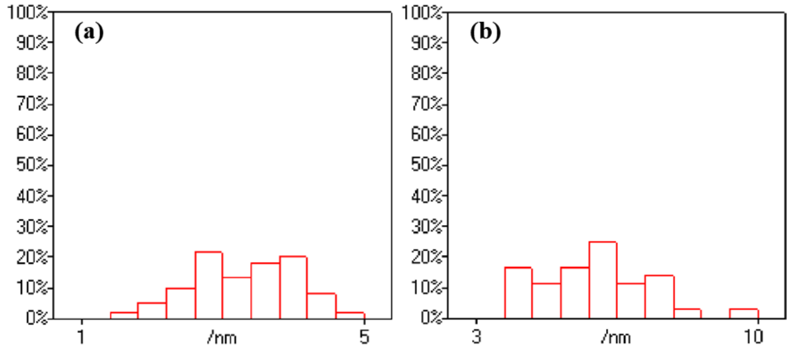


Fig. S2. Particle size distribution of **a,** Fresh Mo@NGn-$CH_4$ and **b,** $Mo_2C$@$SiO_2$-$CH_4$.

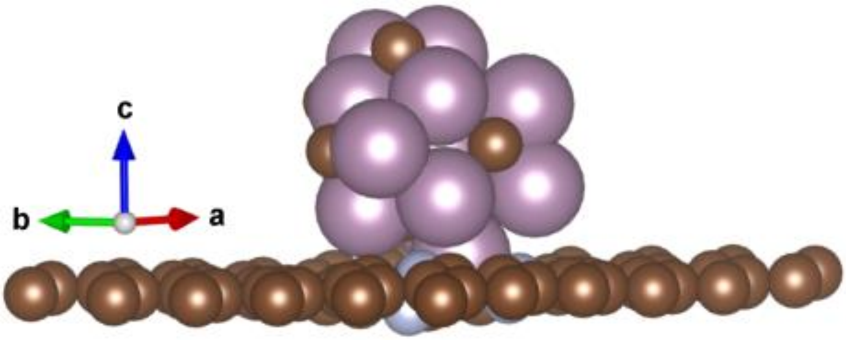


(a)

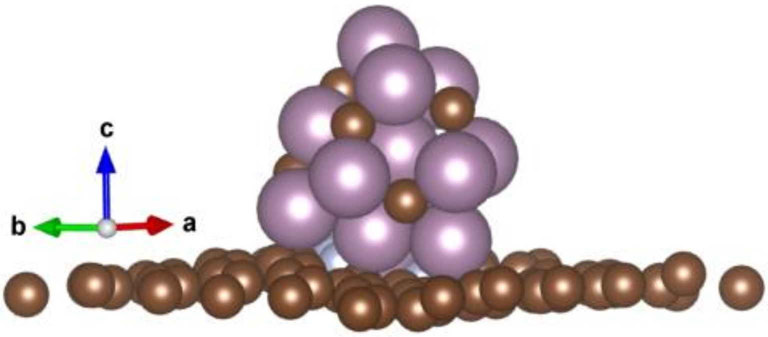


(b)

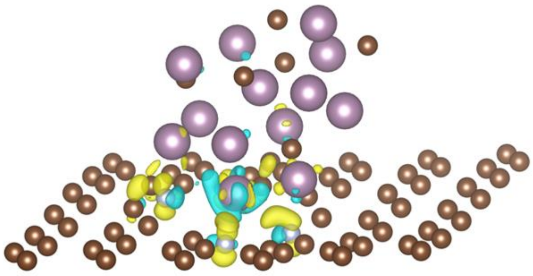

| Component | $Mo_2C$(cluster) | NGn | $Mo_2C$@NGn | Binding energy |
|---|---|---|---|---|
| **Energy (eV)** | -163.47 | -639.66 | -813.88 | 10.75 |

(c)

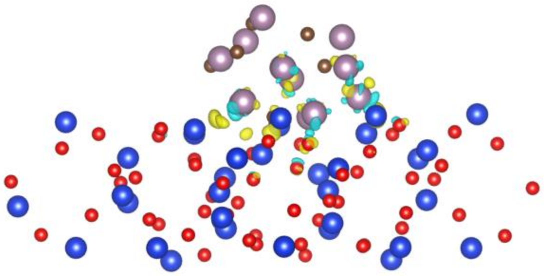

(d)

Fig. S3. (a) The optimized structure of $Mo_2C$@NGn, (b) final structure after 7ps AIMD modelling. Differential electron density of (c) $Mo_2C$@NGn and (d) $Mo_2C$@$SiO_2$ shown with an electronic density isosurface of 0.015e/Å$^3$, where yellow colour denotes electron enrichment and cyan colour denotes electron loss.

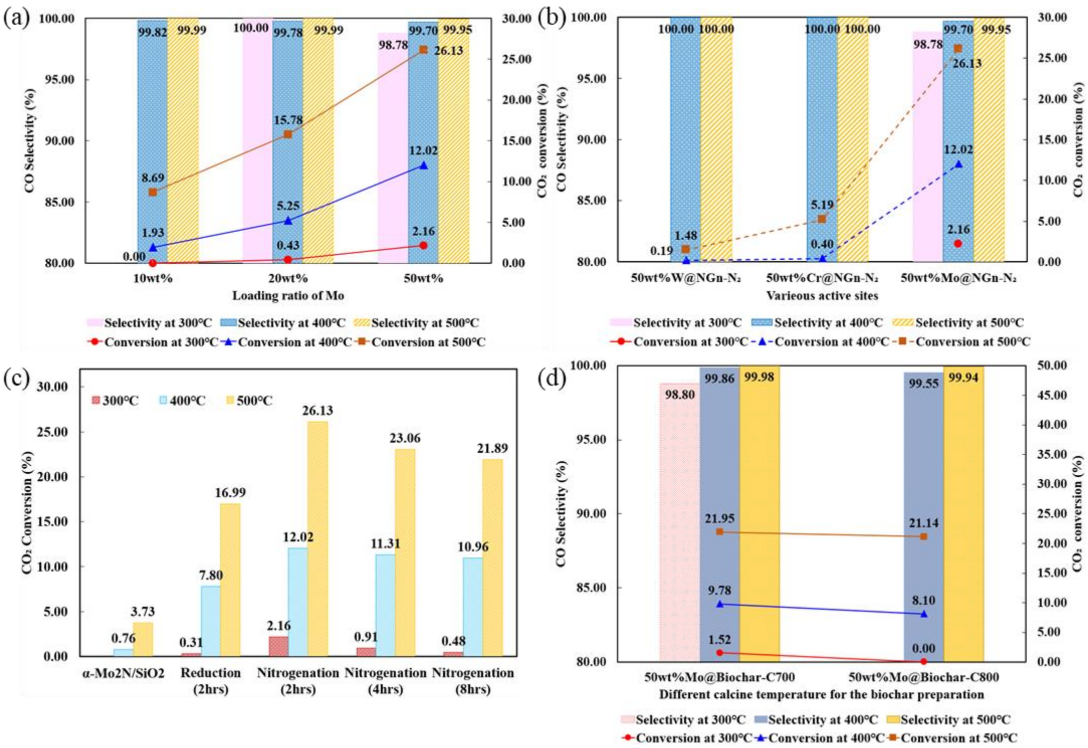


Fig. S4. The catalytic activity of Mo based catalysts pretreated under nitrogenation and reduction. The effects of (a) Loading ratio of Mo (b) metal species (c) preparation methods (d) calcination temperatures for NBiochar support under RWGS reaction conditions (GHSV = 140000ml/g/h, $mol_{CO2}$:$mol_{H2}$=1:2).

| Fukui (-) | | | |
|---|---|---|---|
| Element | atom number | Mulliken | Hirshfeld |
| C ( | 1) | 0.007 | 0.007 |
| C ( | 2) | 0.007 | 0.007 |
| C ( | 3) | 0.007 | 0.007 |
| C ( | 4) | 0.008 | 0.008 |
| C ( | 5) | 0.007 | 0.007 |
| C ( | 6) | 0.008 | 0.008 |
| C ( | 7) | 0.007 | 0.007 |
| C ( | 8) | 0.008 | 0.008 |
| C ( | 9) | 0.007 | 0.007 |
| C ( | 10) | 0.008 | 0.008 |
| C ( | 11) | 0.007 | 0.007 |
| C ( | 12) | 0.007 | 0.007 |
| C ( | 13) | 0.007 | 0.007 |
| C ( | 14) | 0.007 | 0.007 |
| C ( | 15) | 0.007 | 0.007 |
| C ( | 16) | 0.007 | 0.007 |
| C ( | 17) | 0.006 | 0.007 |
| C ( | 18) | 0.007 | 0.006 |
| C ( | 19) | 0.006 | 0.007 |
| C ( | 20) | 0.011 | 0.008 |
| C ( | 21) | 0.007 | 0.007 |
| C ( | 22) | 0.006 | 0.007 |
| C ( | 23) | 0.008 | 0.007 |
| C ( | 24) | 0.007 | 0.007 |
| C ( | 25) | 0.008 | 0.007 |
| C ( | 26) | 0.007 | 0.007 |
| C ( | 27) | 0.006 | 0.007 |
| C ( | 28) | 0.007 | 0.007 |
| C ( | 29) | 0.007 | 0.006 |
| C ( | 30) | 0.005 | 0.006 |
| C ( | 31) | 0.011 | 0.009 |

| Fukui (-) | | | |
|---|---|---|---|
| Element | atom number | Mullike n | Hirshfel d |
| C ( | 32) | 0.007 | 0.007 |
| C ( | 33) | 0.007 | 0.007 |
| C ( | 34) | 0.008 | 0.007 |
| C ( | 35) | 0.009 | 0.008 |
| C ( | 36) | 0.007 | 0.007 |
| C ( | 37) | 0.007 | 0.007 |
| C ( | 38) | 0.008 | 0.006 |
| C ( | 39) | 0.007 | 0.006 |
| C ( | 40) | 0.010 | 0.008 |
| C ( | 41) | 0.006 | 0.007 |
| C ( | 42) | 0.008 | 0.008 |
| C ( | 43) | 0.006 | 0.007 |
| C ( | 44) | 0.008 | 0.008 |
| C ( | 45) | 0.007 | 0.007 |
| C ( | 46) | 0.009 | 0.008 |
| C ( | 47) | 0.007 | 0.007 |
| C ( | 48) | 0.008 | 0.007 |
| C ( | 49) | 0.011 | 0.008 |
| C ( | 50) | 0.008 | 0.008 |
| C ( | 51) | 0.012 | 0.009 |
| C ( | 52) | 0.009 | 0.007 |
| C ( | 53) | 0.008 | 0.007 |
| C ( | 54) | 0.008 | 0.007 |
| C ( | 55) | 0.005 | 0.006 |
| C ( | 56) | 0.008 | 0.008 |
| C ( | 57) | 0.007 | 0.007 |
| C ( | 58) | 0.008 | 0.007 |
| C ( | 59) | 0.006 | 0.007 |
| C ( | 60) | 0.009 | 0.009 |
| C ( | 61) | 0.007 | 0.007 |
| C ( | 62) | 0.008 | 0.007 |

| Fukui (-) | | | |
|---|---|---|---|
| Element | atom number | Mullike n | Hirshfel d |
| C ( | 63) | 0.008 | 0.007 |
| C ( | 64) | 0.006 | 0.007 |
| C ( | 65) | 0.008 | 0.007 |
| C ( | 66) | 0.006 | 0.007 |
| C ( | 67) | 0.001 | 0.009 |
| C ( | 68) | 0.002 | 0.009 |
| C ( | 69) | 0.001 | 0.002 |
| C ( | 70) | 0.015 | 0.017 |
| C ( | 71) | 0.011 | 0.013 |
| C ( | 72) | 0.026 | 0.025 |
| N ( | 73) | 0.004 | 0.005 |
| N ( | 74) | 0.003 | 0.006 |
| N ( | 75) | 0.009 | 0.010 |
| N ( | 76) | 0.008 | 0.010 |
| Mo( | 77) | 0.014 | 0.009 |
| Mo( | 78) | 0.025 | 0.024 |
| Mo( | 79) | 0.000 | 0.009 |
| Mo( | 80) | 0.002 | 0.001 |
| Mo( | 81) | 0.050 | 0.051 |
| Mo( | 82) | 0.027 | 0.033 |
| Mo( | 83) | 0.007 | 0.011 |
| Mo( | 84) | 0.015 | 0.019 |
| Mo( | 85) | 0.058 | 0.054 |
| Mo( | 86) | 0.044 | 0.042 |
| Mo( | 87) | 0.018 | 0.016 |
| Mo( | 88) | 0.080 | 0.072 |
| Mo( | 89) | 0.049 | 0.043 |
| Mo( | 90) | 0.006 | 0.008 |
| Mo( | 91) | 0.027 | 0.023 |
| | | | |
| | | | |

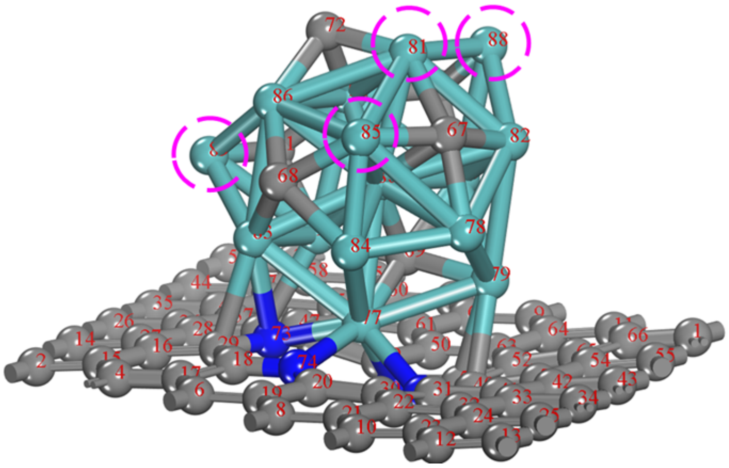


Fig. S5. Fukui analysis of an intact cluster of $Mo_2C$ supported on NGn.

| Fukui (-) | | | |
|---|---|---|---|
| Element | atom number | Mulliken | Hirshfeld |
| C ( | 1) | 0.009 | 0.008 |
| C ( | 2) | 0.007 | 0.007 |
| C ( | 3) | 0.007 | 0.007 |
| C ( | 4) | 0.007 | 0.007 |
| C ( | 5) | 0.007 | 0.007 |
| C ( | 6) | 0.008 | 0.008 |
| C ( | 7) | 0.008 | 0.008 |
| C ( | 8) | 0.007 | 0.008 |
| C ( | 9) | 0.008 | 0.008 |
| C ( | 10) | 0.008 | 0.008 |
| C ( | 11) | 0.008 | 0.008 |
| C ( | 12) | 0.007 | 0.007 |
| C ( | 13) | 0.008 | 0.008 |
| C ( | 14) | 0.007 | 0.007 |
| C ( | 15) | 0.008 | 0.008 |
| C ( | 16) | 0.007 | 0.007 |
| C ( | 17) | 0.008 | 0.008 |
| C ( | 18) | 0.006 | 0.006 |
| C ( | 19) | 0.007 | 0.008 |
| C ( | 20) | 0.01 | 0.007 |
| C ( | 21) | 0.008 | 0.008 |
| C ( | 22) | 0.006 | 0.007 |
| C ( | 23) | 0.008 | 0.008 |
| C ( | 24) | 0.007 | 0.007 |
| C ( | 25) | 0.009 | 0.008 |
| C ( | 26) | 0.007 | 0.008 |
| C ( | 27) | 0.007 | 0.008 |
| C ( | 28) | 0.007 | 0.007 |
| C ( | 29) | 0.008 | 0.006 |
| C ( | 30) | 0.005 | 0.006 |
| C ( | 31) | 0.013 | 0.01 |

| Fukui (-) | | | |
|---|---|---|---|
| Element | atom number | Mullike n | Hirshfel d |
| C ( | 32) | 0.008 | 0.007 |
| C ( | 33) | 0.007 | 0.007 |
| C ( | 34) | 0.008 | 0.008 |
| C ( | 35) | 0.009 | 0.008 |
| C ( | 36) | 0.008 | 0.008 |
| C ( | 37) | 0.007 | 0.007 |
| C ( | 38) | 0.011 | 0.008 |
| C ( | 39) | 0.007 | 0.006 |
| C ( | 40) | 0.012 | 0.008 |
| C ( | 41) | 0.007 | 0.007 |
| C ( | 42) | 0.008 | 0.008 |
| C ( | 43) | 0.006 | 0.007 |
| C ( | 44) | 0.008 | 0.008 |
| C ( | 45) | 0.007 | 0.007 |
| C ( | 46) | 0.009 | 0.008 |
| C ( | 47) | 0.007 | 0.007 |
| C ( | 48) | 0.007 | 0.007 |
| C ( | 49) | 0.012 | 0.009 |
| C ( | 50) | 0.007 | 0.007 |
| C ( | 51) | 0.012 | 0.009 |
| C ( | 52) | 0.009 | 0.008 |
| C ( | 53) | 0.01 | 0.008 |
| C ( | 54) | 0.008 | 0.007 |
| C ( | 55) | 0.006 | 0.007 |
| C ( | 56) | 0.008 | 0.008 |
| C ( | 57) | 0.008 | 0.008 |
| C ( | 58) | 0.008 | 0.008 |
| C ( | 59) | 0.007 | 0.007 |
| C ( | 60) | 0.009 | 0.008 |
| C ( | 61) | 0.006 | 0.007 |
| C ( | 62) | 0.008 | 0.008 |

| Fukui (-) | | | |
|---|---|---|---|
| Element | atom number | Mullike n | Hirshfel d |
| C ( | 63) | 0.008 | 0.008 |
| C ( | 64) | 0.006 | 0.007 |
| C ( | 65) | 0.009 | 0.007 |
| C ( | 66) | 0.006 | 0.007 |
| C ( | 67) | 0.003 | 0.011 |
| C ( | 68) | 0.004 | 0.01 |
| C ( | 69) | 0.002 | 0.002 |
| C ( | 70) | 0.023 | 0.025 |
| C ( | 71) | 0.015 | 0.018 |
| C ( | 72) | 0.012 | 0.019 |
| N ( | 73) | 0.003 | 0.006 |
| N ( | 74) | 0.003 | 0.005 |
| N ( | 75) | 0.008 | 0.01 |
| N ( | 76) | 0.008 | 0.01 |
| Mo( | 77) | 0.02 | 0.011 |
| Mo( | 78) | 0.023 | 0.023 |
| Mo( | 79) | 0 | 0.009 |
| Mo( | 80) | -0.01 | 0.002 |
| Mo( | 81) | 0.049 | 0.048 |
| Mo( | 82) | 0.015 | 0.017 |
| Mo( | 83) | 0.011 | 0.016 |
| Mo( | 84) | 0.087 | 0.07 |
| Mo( | 85) | 0.014 | 0.019 |
| Mo( | 86) | 0.084 | 0.074 |
| Mo( | 87) | 0.065 | 0.053 |
| Mo( | 88) | 0.001 | 0.01 |
| Mo( | 89) | 0.041 | 0.037 |
| | | | |
| | | | |
| | | | |
| | | | |

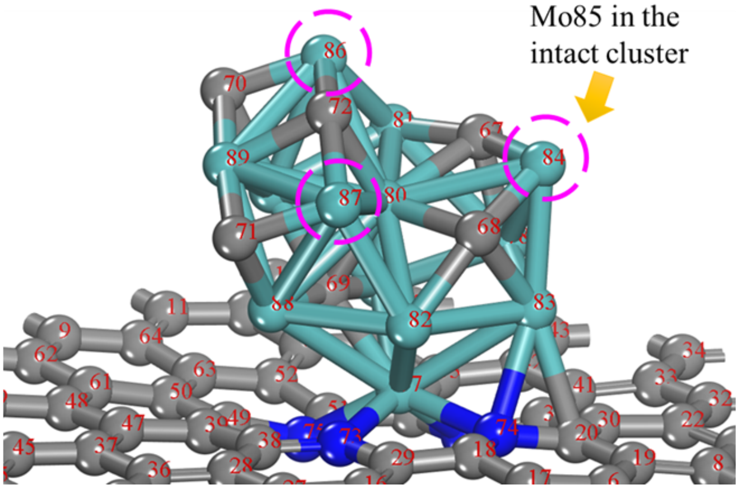


Fig. S6. Fukui analysis of the dual-defected $Mo_2C$_V5&10@NGn.

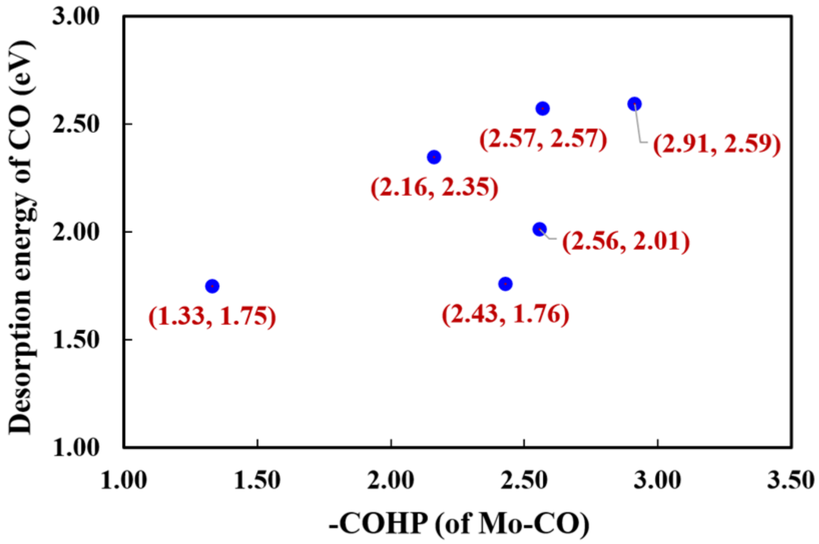


Fig. S7. Independence of CO desorption energy and Mo-CO bond strength in a variety of $Mo_2C$ catalytic system.

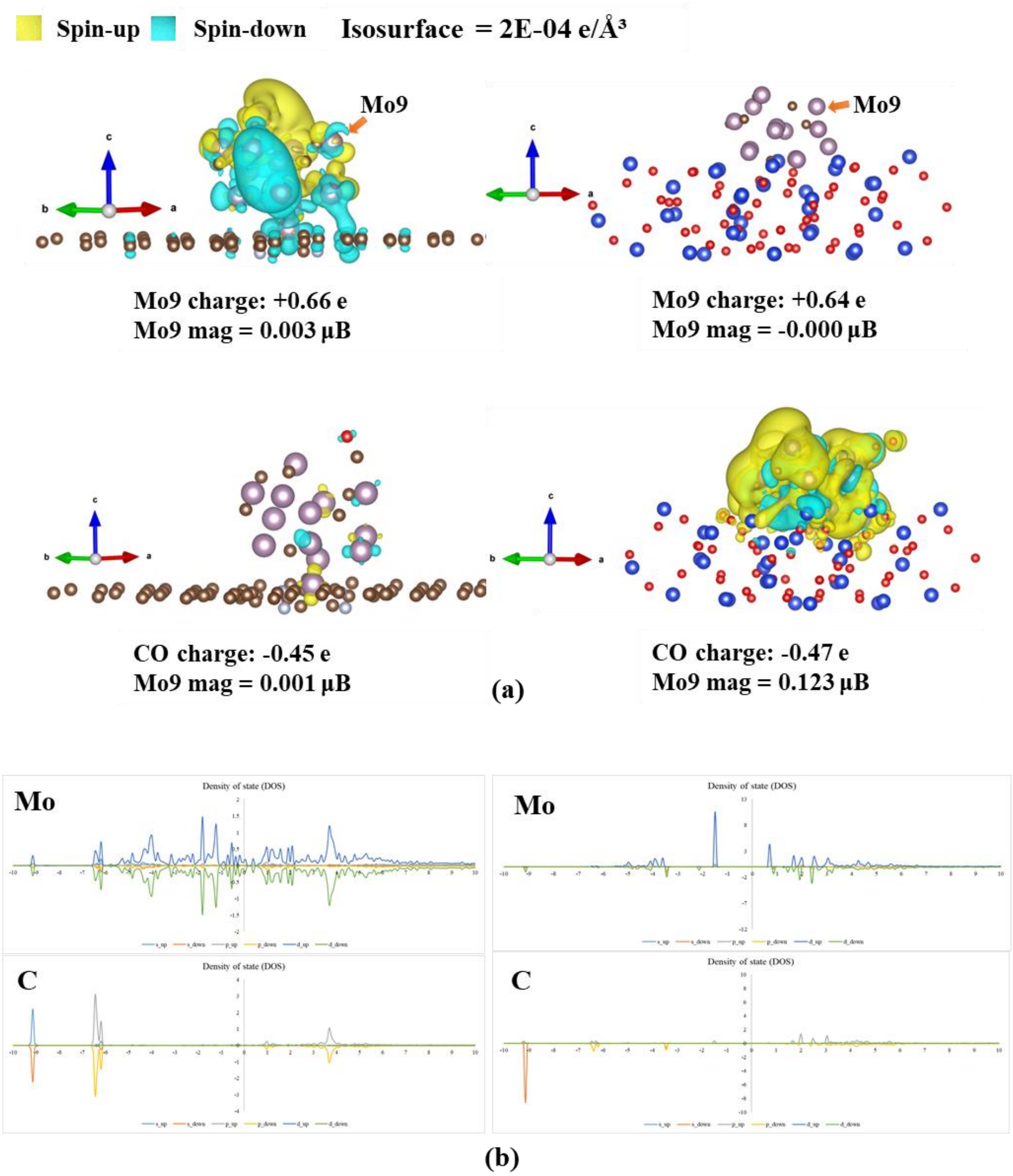


Fig. S8. (a) Electronic spin of $Mo_2C$_V5&10@NGn and $Mo_2C$_V5&10@$SiO_2$ before and after CO adsorption. (b) The corresponding pDOS for Mo9 and C (in CO) in the two catalytic systems.

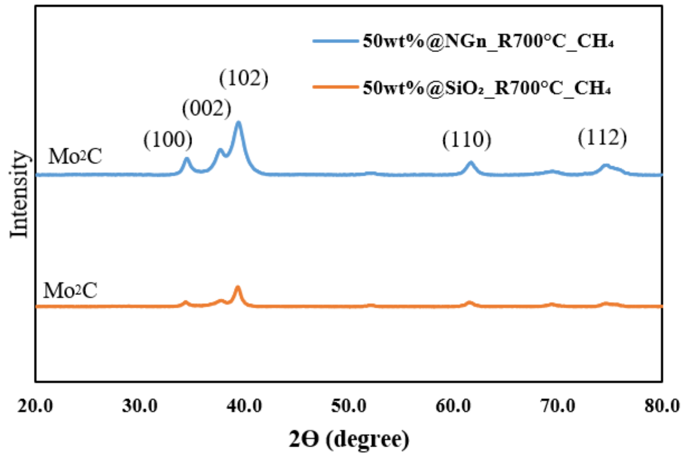


Fig. S9. XRD patterns of the Mo based catalysts over NGn and $SiO_2$ respectively

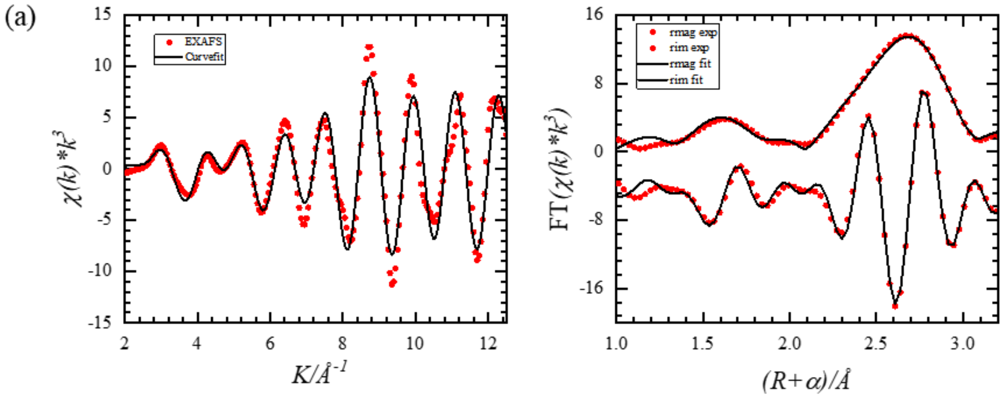


| Path | d/Å | R/Å | N | S0² | σ²/Å |
|---|---|---|---|---|---|
| Mo-C | 2.11 | 2.08 | 3 | 0.74 | 0.003 |
| Mo-Mo | 3.01 | 2.97 | 12 | 0.46 | 0.005 |

(b)

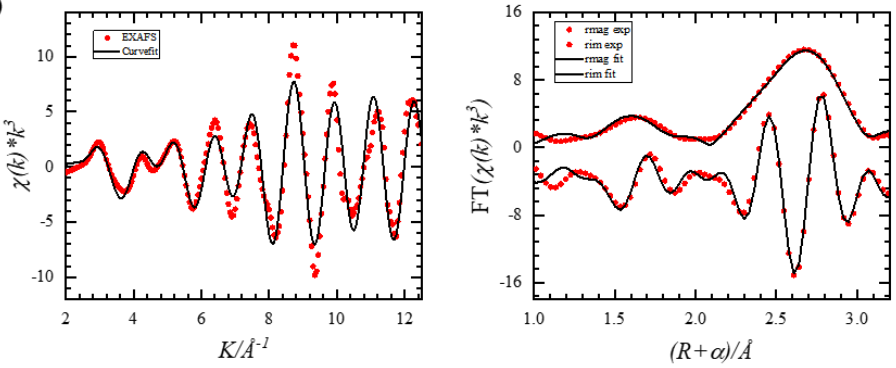


| Path | d/Å | R/Å | N | S0² | σ²/Å |
|---|---|---|---|---|---|
| Mo-C | 2.11 | 2.07 | 3 | 0.76 | 0.004 |
| Mo-Mo | 3.01 | 2.98 | 12 | 0.42 | 0.005 |

(c)

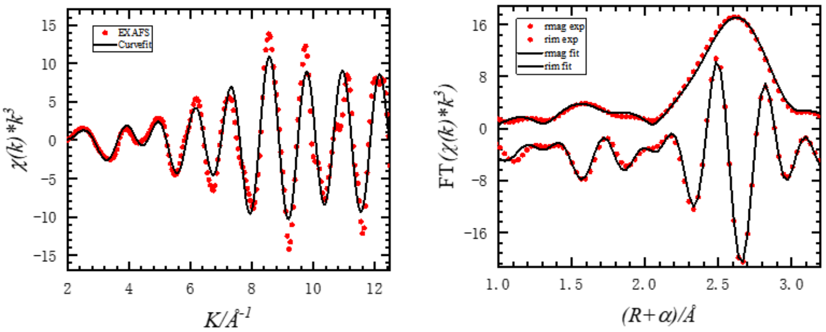


| Path | d/Å | R/Å | N | S0² | σ²/Å |
|---|---|---|---|---|---|
| Mo-C | 2.11 | 2.08 | 3 | 0.67 | 0.002 |
| Mo-Mo | 3.01 | 2.98 | 12 | 0.67 | 0.006 |

(d)

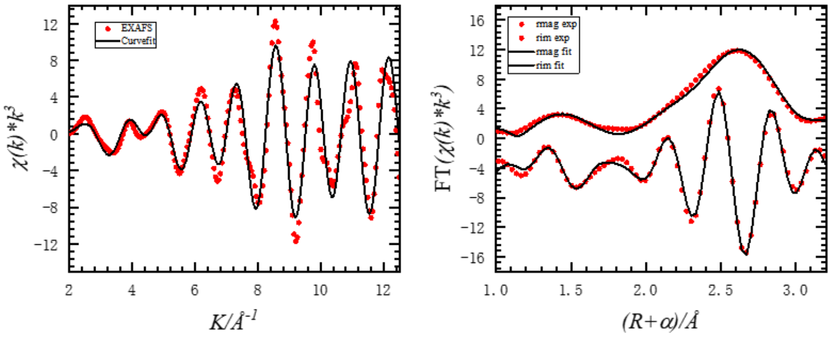


| Path | d/Å | R/Å | N | S0² | σ²/Å |
|---|---|---|---|---|---|
| Mo-C | 2.11 | 2.08 | 3 | 0.69 | 0.001 |
| Mo-Mo | 3.01 | 2.98 | 12 | 0.51 | 0.005 |

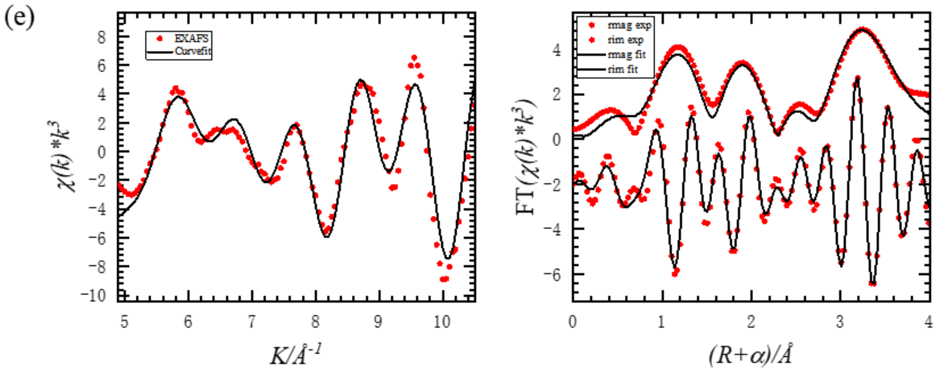


| Path | d/Å | R/Å | N | $SO^2$ | $\sigma^2$/Å |
|---|---|---|---|---|---|
| Mo-O | 1.67 | 1.64 | 3 | 0.87 | 0.008 |
| Mo-O | 2.29 | 2.29 | 3 | 0.87 | 0.003 |
| Mo-Mo | 3.67 | 3.67 | 4 | 0.87 | 0.002 |

Fig. S10. The EXAFS results and curve fittings for Mo based species. (a) Mo K-edge EXAFS (points) and the curvefit (line) for $Mo_2C$@NGn_fresh, shown in k3-weighted k-space and R space. R-factor = 0.0180. (b) Mo K-edge EXAFS (points) and the curvefit (line) for $Mo_2C$@NGn_spent, shown in k3-weighted k-space and R space. R-factor = 0.0288. (c) Mo K-edge EXAFS (points) and the curvefit (line) for $Mo_2C$, shown in k3-weighted k-space and R space. R-factor = 0.0183. (d) Mo K-edge EXAFS (points) and the curvefit (line) for $Mo_2C@SiO_2$, shown in k3-weighted k-space and R space. R-factor = 0.0209. (e) Mo K-edge EXAFS (points) and the curvefit (line) for $MoO_3$, shown in k3-weighted k-space and R space. R-factor = 0.0258. The data are k3-weighted and not phase corrected.

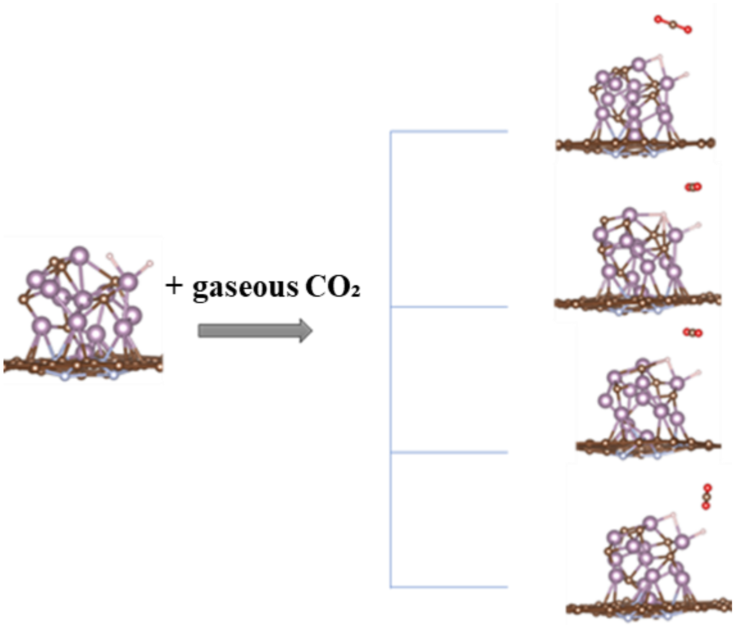


Fig. S11. The plausible catalytic reaction in the ER mechanism

Table S12. TOF of the $Mo_2C$ cluster supported on NGn and Nbiochar (GHSV = 930000ml/g/h, $mol_{CO2}$:$mol_{H2}$=1:2)

| Temperature | TOF (over Mo2C@NGn) | TOF (over Mo2C@NBio) |
|---|---|---|
| 300°C | 9.58 | 4.11 |
| 320°C | 15.33 | 7.23 |
| 340°C | 28.20 | 11.01 |
| 360°C | 47.92 | 17.42 |
| 380°C | 68.73 | 25.96 |
| 400°C | 95.84 | 36.88 |

Note: The data in above table do not reflect the peak reaction rate, as the mass transfer to catalyst surface might be limited at an ultra-high GHSV of 930000 ml/g/h, the reaction rate estimated in the article was based on test with a GHSV of 140000ml/g/h which we think would reflect the best performance of this catalyst.

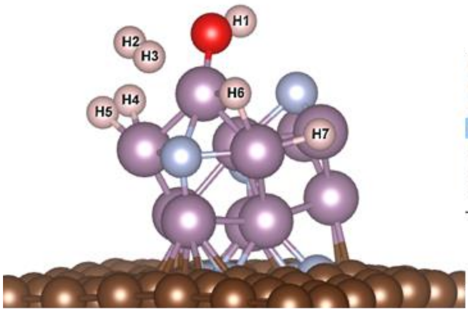


| | | | | | | |
|---|---|---|---|---|---|---|
| 86 | 4.266900 | 5.365050 | 11.216209 | 0.381422 | 0.109823 | 281.093763 |
| 87 | 4.428147 | 0.939629 | 10.692235 | 0.986651 | 0.280347 | 334.076127 |
| 88 | 5.025410 | 1.157501 | 10.287404 | 1.023313 | 0.319454 | 93.351119 |
| 89 | 1.507868 | 3.530113 | 8.878271 | 1.004335 | 0.327250 | 75.212948 |
| 90 | 1.071238 | 2.875051 | 8.535815 | 1.138495 | 0.380712 | 123.312767 |
| 91 | 6.227451 | 3.431217 | 9.214536 | 1.377868 | 0.580548 | 106.707243 |
| 92 | 7.519986 | 5.577437 | 8.021672 | 1.453272 | 0.636129 | 109.940032 |

VACUUM CHARGE: 0.0000
VACUUM VOLUME: 0.0000
NUMBER OF ELECTRONS: 466.0000

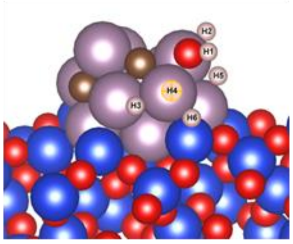


| | | | | | | |
|---|---|---|---|---|---|---|
| 111 | 7.032545 | 2.243087 | 10.191516 | 0.382579 | 0.078808 | 45.639220 |
| 112 | 7.359294 | 4.926348 | 10.556730 | 1.356304 | 0.495180 | 159.177184 |
| 113 | 3.290970 | 1.884760 | 8.013931 | 1.356305 | 0.602772 | 27.459730 |
| 114 | 4.960145 | 0.881686 | 8.796210 | 1.266923 | 0.522588 | 75.144381 |
| 115 | 7.650913 | 4.096308 | 8.531448 | 1.339780 | 0.563984 | 26.637910 |
| 116 | 5.868388 | 1.242052 | 7.286075 | 1.503404 | 0.581659 | 16.896323 |

VACUUM CHARGE: 0.0000
VACUUM VOLUME: 0.0000
NUMBER OF ELECTRONS: 686.0000

Fig. S13. Charge analysis of surface *H atoms on $Mo_2N$_vMo@NGn and $Mo_2C$_vMo@$SiO_2$.

Table S14. Physical properties of the sample 50%Mo@NGn.

| Parameters | Values |
|---|---|
| Diameter | 2-10 μm |
| Special surface area (BET) | 40.0 $m^2/g$ |
| Average pore volume | 0.24 $cm^3/g$ |
| Average pore size | 23.87 nm |
| Thickness | 1～3 nm |
| Mo content | 48.5 wt% |
| Nitrogen content in NGn | 5.38 wt% |

Table S15. The imaginary frequency of the transition state structures for each elementary reaction shown in Fig. 7 (a) and (b)

| TS No. | The unique imaginary frequency ($cm^{-1}$) |
|---|---|
| TS1-1 | -302.74 |
| TS1-2 | -1394.46 |
| TS1-3 | -1247.33 |
| TS1-4 | -1336.34 |
| TS2-1 | -1403.72 |
| TS2-2 | -1346.58 |
| TS3-1 | -822.08 |
| TS4-1 | -401.61 |
| TS5-1 | -1220.22 |
| TS5-2 | -159.14 |
| TS6-1 | -1226.76 |
| TS6-1 | -1473.37 |

Table S16. Reaction tests over 1%W@NGn in comparison with Mo based catalyst

| Catalyst | Temperature | Conversion of $CO_2$ (%) | CO yield (conc.%) |
|---|---|---|---|
| **1%W@NGn** | 400°C | 0.25 | 0.05 |
| **1%W@NGn** | 500°C | 0.27 | 0.06 |
| **Mo based catalyst** | 500°C | 30.5 | 8.53 |

Fig. S17. Independence tests for (a) cut-off energy and (b) k-points respectively

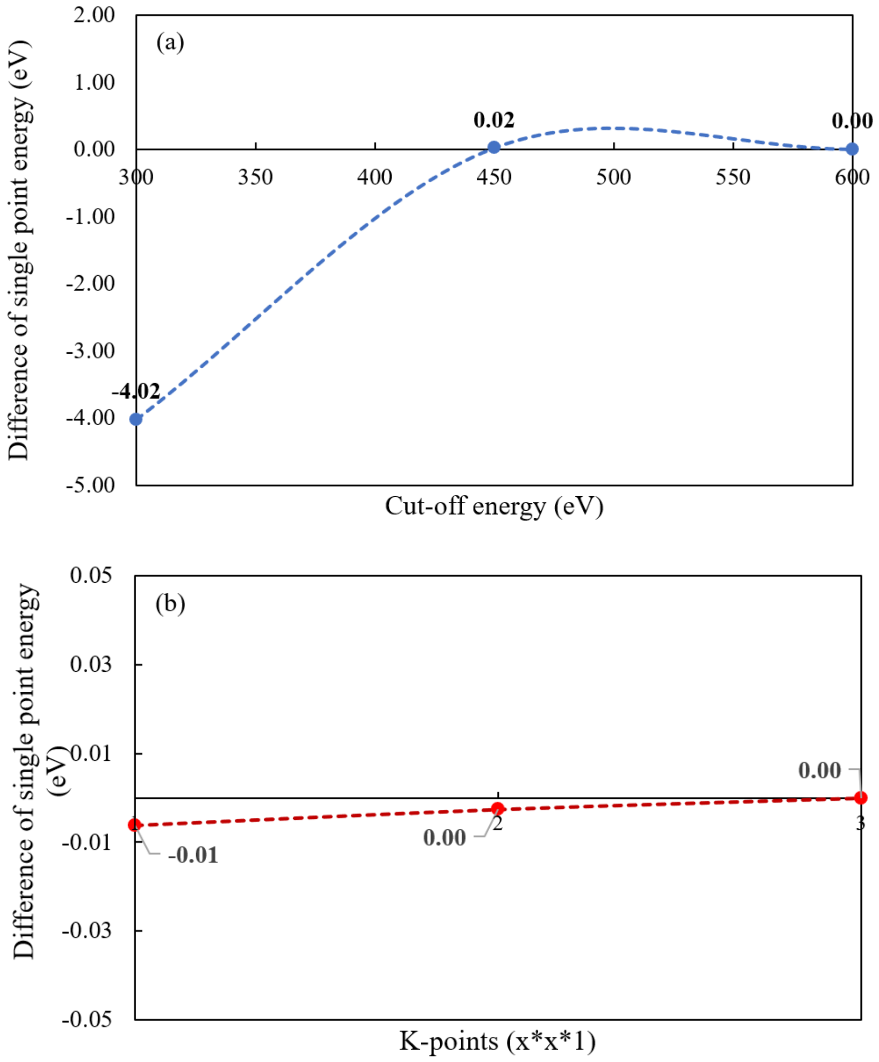

Table S18. Validation of the present computational method

| **Lattice parameters** | **Experiment** [19,20] | **Computation (this work)** |
| --- | --- | --- |
| 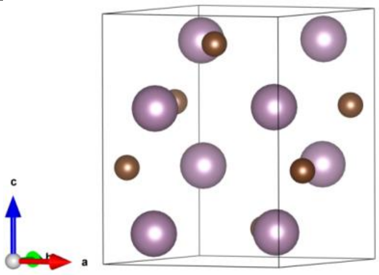 | a=β=γ=90º | a=β=γ=90º |
| | a= 4.74 Å<br>b= 5.21 Å<br>c= 6.03 Å | a= 4.71647 Å<br>b= 5.19692 Å<br>c= 6.02343 Å |
| 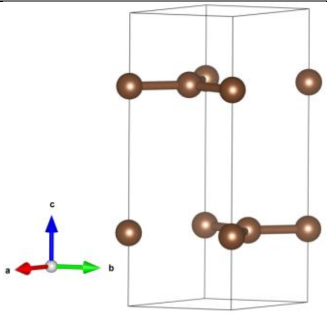 | a=β= 90º<br>γ= 120º | a=β= 90º<br>γ= 120º |
| | a=b= 2.47 Å<br>c= 6.79 Å | a=b= 2.46594 Å<br>c= 6.81282 Å |

**Reference:**


(1) Porosoff, M. D.; Yang, X.; Boscoboinik, J. A.; Chen, J. G. Molybdenum Carbide as Alternative Catalysts to Precious Metals for Highly Selective Reduction of $CO_2$ to CO. *Angewandte Chemie International Edition* **2014**, *53* (26), 6705–6709. https://doi.org/10.1002/anie.201404109.

(2) Li, Y.; Zhao, Z.; Lu, W.; Zhu, H.; Sun, F.; Mei, B.; Jiang, Z.; Lyu, Y.; Chen, X.; Guo, L.; Wu, T.; Ma, X.; Meng, Y.; Ding, Y. Single-Atom Co-N-C Catalysts for High-Efficiency Reverse Water-Gas Shift Reaction. *Applied Catalysis B: Environmental* **2023**, *324* (November 2022), 122298. https://doi.org/10.1016/j.apcatb.2022.122298.

(3) Du, P.; Qi, R.; Zhang, Y.; Gu, Q.; Xu, X.; Tan, Y.; Liu, X.; Wang, A.; Zhu, B.; Yang, B.; Zhang, T. Single-Atom-Driven Dynamic Carburization over $Pd_1$–$FeO_x$ Catalyst Boosting $CO_2$ Conversion. *Chem* **2022**, *8* (12), 3252–3262. https://doi.org/10.1016/j.chempr.2022.08.012.

(4) Mao, D.; Zhang, J.; Zhang, H.; Wu, D. A Highly Efficient Cu-ZnO/SBA-15 Catalyst for $CO_2$ Hydrogenation to CO under Atmospheric Pressure. *Catalysis Today* **2022**, *402* (November 2021), 60–66. https://doi.org/10.1016/j.cattod.2022.03.002.

(5) Zhang, R.; Wang, X.; Wang, K.; Wang, H.; Liu, L.; Wu, X.; Geng, B.; Chu, X.; Song, S.; Zhang, H. Synergism of Ultrasmall Pt Clusters and Basic $La_2O_2CO_3$ Supports Boosts the Reverse Water Gas Reaction Efficiency. *Advanced Energy Materials* **2023**, *2203806*, 2203806. https://doi.org/10.1002/aenm.202203806.

(6) Nityashree, N.; Price, C. A. H.; Pastor-Perez, L.; Manohara, G. V.; Garcia, S.; Maroto-Valer, M. M.; Reina, T. R. Carbon Stabilised Saponite Supported Transition Metal-Alloy Catalysts for Chemical $CO_2$ Utilisation via Reverse Water-Gas Shift Reaction. *Applied Catalysis B: Environmental* **2020**, *261*, 118241. https://doi.org/10.1016/j.apcatb.2019.118241.

(7) Pastor-Pérez, L.; Baibars, F.; Le Sache, E.; Arellano-García, H.; Gu, S.; Reina, T. R. $CO_2$ Valorisation via Reverse Water-Gas Shift Reaction Using Advanced Cs Doped Fe-Cu/$Al_2O_3$ Catalysts. *Journal of $CO_2$ Utilization* **2017**, *21* (July), 423–428. https://doi.org/10.1016/j.jcou.2017.08.009.

(8) Porosoff, M. D.; Chen, J. G. Trends in the Catalytic Reduction of $CO_2$ by Hydrogen over Supported Monometallic and Bimetallic Catalysts. *Journal of Catalysis* **2013**, *301*, 30–37. https://doi.org/10.1016/j.jcat.2013.01.022.

(9) Porosoff, M. D.; Baldwin, J. W.; Peng, X.; Mpourmpakis, G.; Willauer, H. D. Potassium-Promoted Molybdenum Carbide as a Highly Active and Selective Catalyst for $CO_2$ Conversion to CO. *ChemSusChem* **2017**, *10* (11), 2408–2415. https://doi.org/10.1002/cssc.201700412.

(10) Dalebout, R.; Barberis, L.; Visser, N. L.; van der Hoeven, J. E. S.; van der Eerden, A. M. J.; Stewart, J. A.; Meirer, F.; de Jong, K. P.; de Jongh, P. E. Manganese Oxide as a Promoter for Copper Catalysts in $CO_2$ and CO Hydrogenation. *ChemCatChem* **2022**, *14* (19). https://doi.org/10.1002/cctc.202200451.

(11) Zhang, R.; Wei, A.; Zhu, M.; Wu, X.; Wang, H.; Zhu, X.; Ge, Q. Tuning Reverse Water Gas Shift and Methanation Reactions during $CO_2$ Reduction on Ni Catalysts via Surface Modification by $MoO_x$. *Journal of $CO_2$ Utilization* **2021**, *52*, 101678. https://doi.org/10.1016/j.jcou.2021.101678.

(12) Zhou, H.; Chen, Z.; Kountoupi, E.; Tsoukalou, A.; Abdala, P. M.; Florian, P.; Fedorov, A.; Müller, C. R. Two-Dimensional Molybdenum Carbide 2D-$Mo_2C$ as a Superior Catalyst for $CO_2$ Hydrogenation. *Nature Communications* **2021**, *12* (1), 5510. https://doi.org/10.1038/s41467-021-25784-0.

(13) Rutherford, B.; Panaritis, C.; Pahija, E.; Couillard, M.; Patarachao, B.; Bensebaa, F.; Shadbahr, J.; Patience, G. S.; Boffito, D. C. Ni Nanoparticles on $Co_3O_4$ Catalyze the

Reverse Water-Gas Shift with 95% Co Selectivity at 300 °C. *SSRN Electronic Journal* **2022**, *348* (May), 128523. https://doi.org/10.2139/ssrn.4267683.

(14) Liu, X.; Kunkel, C.; Ramírez de la Piscina, P.; Homs, N.; Viñes, F.; Illas, F. Effective and Highly Selective CO Generation from $CO_2$ Using a Polycrystalline α-$Mo_2C$ Catalyst. *ACS Catalysis* **2017**, *7* (7), 4323–4335. https://doi.org/10.1021/acscatal.7b00735.

(15) Spennati, E.; Garbarino, G.; Savio, L.; Vattuone, L.; Riani, P.; Busca, G. $CO_2$ Methanation vs Reverse WGS Activity on Co/γ-$Al_2O_3$ Catalysts at Atmospheric Pressure: Effect of Cobalt Loading and Silica Addition on Selectivity and Stability. *Catalysis Today* **2023**, *420* (December 2022), 114164. https://doi.org/10.1016/j.cattod.2023.114164.

(16) Nejadsalim, A.; Bashiri, N.; Godini, H. R.; Oliveira, R. L.; Tufail Shah, A.; Bekheet, M. F.; Thomas, A.; Schomäcker, R.; Gurlo, A.; Görke, O. Core-Sheath Pt-$CeO_2$/Mesoporous $SiO_2$ Electrospun Nanofibers as Catalysts for the Reverse Water Gas Shift Reaction. *Nanomaterials* **2023**, *13* (3), 485. https://doi.org/10.3390/nano13030485.

(17) Morse, J. R.; Juneau, M.; Baldwin, J. W.; Porosoff, M. D.; Willauer, H. D. Alkali Promoted Tungsten Carbide as a Selective Catalyst for the Reverse Water Gas Shift Reaction. *Journal of $CO_2$ Utilization* **2020**, *35* (August 2019), 38–46. https://doi.org/10.1016/j.jcou.2019.08.024.

(18) Zhang, Q.; Pastor-Pérez, L.; Jin, W.; Gu, S.; Reina, T. R. Understanding the Promoter Effect of Cu and Cs over Highly Effective β-$Mo_2C$ Catalysts for the Reverse Water-Gas Shift Reaction. *Applied Catalysis B: Environmental* **2019**, *244* (December 2018), 889–898. https://doi.org/10.1016/j.apcatb.2018.12.023.

(19) Jette, E. R.; Foote, F. Precision Determination of Lattice Constants. *The Journal of Chemical Physics* **1935**, *3* (10), 605–616. https://doi.org/10.1063/1.1749562.

(20) Hassel, O.; Mark, H. Über Die Kristallstruktur Des Graphits. *Zeitschrift für Physik* **1924**, *25*, 317–337.
